# Modulation of Peptoid Nanostructure for Antibiofilm Hydrogel Interfaces

**DOI:** 10.1021/acs.nanolett.5c05266

**Published:** 2026-02-26

**Authors:** Jae Won Yun, Il-Soo Park, Heewoong Yoon, Jiwon Woo, Dong-Yeong Kim, Woojin Yang, Jieun Choi, Dal-Hee Min, Jung-Hyun Lee, Jiwon Seo, Jae Hong Kim

**Affiliations:** † Convergence Research Center for Solutions to Electromagnetic Interference in Future-mobility, Korea Institute of Science and Technology (KIST), Seoul 02792, Republic of Korea; ‡ Electronic and Hybrid Materials Research Center, Korea Institute of Science and Technology (KIST), Seoul 02792, Republic of Korea; § Department of Chemistry, Gwangju Institute of Science and Technology (GIST), Gwangju 61005, Republic of Korea; ∥ Department of Chemistry, 26725 Seoul National University, Seoul 08826, Republic of Korea; ⊥ Department of Chemical and Biological Engineering, Korea University, Seoul 02841, Republic of Korea; # GIST InnoCORE AI-Nano Convergence Institute for Early Detection of Neurodegenerative Diseases, 65419 Gwangju Institute of Science and Technology, Gwangju 61005, Republic of Korea

**Keywords:** peptoid, hydrogel coating, multifunctional, antimicrobial, antiadhesive, antibiofilm

## Abstract

Most hospital-acquired infections originate from bacterial biofilms on implantable devices, where the extracellular polymeric substance (EPS) matrix protects microbes and promotes multidrug resistance (MDR). Preventing biofilm initiation, particularly bacterial adhesion and proliferation, offers an effective strategy to combat device-associated infections. However, developing biocompatible materials that combine antimicrobial and antiadhesive functions remains a challenge. Herein, we present a strategy to engineer antibiofilm hydrogels by incorporating antimicrobial peptoids (ampetoids) into a gelatin-based matrix with controlled supramolecular organization. By tuning the stoichiometric ratio between thiol-functionalized ampetoids and norbornene groups in the matrix, we controlled peptoid nanostructure within the hydrogel, where suppression of peptoid self-assembly proved critical for balancing antimicrobial activity, antiadhesive properties, and cytocompatibility. This molecular-level control enabled the hydrogels to inhibit biofilm formation by *S. aureus* and *P. aeruginosa*. These results highlight regulation of peptoid self-assembly within hydrogels as a promising approach for designing multifunctional antibiofilm coatings for contamination-prone medical devices.

## Graphical abstract

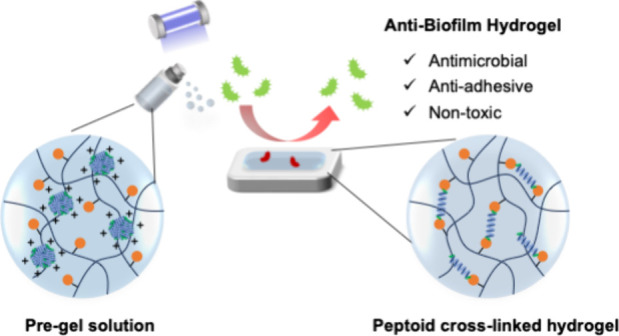

Bacterial biofilms, surface-attached microbial communities encased within a self-secreted extracellular polymeric substance (EPS) matrix, represent the predominant mode of bacterial existence in natural environments rather than free-floating planktonic cells.^1,2^ Biofilms are implicated in over 80% of chronic infections and account for 60–70% of hospital-acquired infections, particularly those associated with implantable medical devices such as catheters, stents, and ventilators.^3–6^ The dense EPS matrix not only restricts antibiotic penetration but also creates a harsh microenvironment,^7,8^ rendering biofilm-embedded bacteria up to 10–1000 times more resistant to antibiotics than their planktonic counterparts.^9,10^ Consequently, biofilm-associated infections often necessitate repeated, high-dose antibiotic treatments, which paradoxically accelerate the emergence and spread of multidrug-resistant (MDR) bacteria amid stagnation in antibiotic development.^11^ These challenges highlight the urgent need for material-based surface engineering strategies capable of simultaneously preventing MDR pathogen adhesion and biofilm formation.

Strategies to inhibit biofilm formation primarily target two critical stages: preventing initial bacterial adhesion and eradicating attached bacteria. Antiadhesive coatings, such as those based on hydrophilic or superhydrophobic polymers (e.g., polyethylene glycol or zwitterionic polymers), create hydration or air layers to reduce surface energy and minimize bacterial attachment.^12–16^ However, their protection is often transient, as these materials fail to prevent colonization by already-adhered bacteria.^17–19^ Antibacterial coatings, by contrast, aim to eliminate surface-bound bacteria through immobilization or release of antimicrobial agents.^20^ To overcome antimicrobial resistance (AMR), alternative agents, including inorganic nanomaterials,^21^ quaternary ammonium compounds (QACs),^22^ and antimicrobial peptides (AMPs)^23,24^ have been explored. Although these agents display broad antimicrobial activity, challenges such as toxicity, suboptimal efficacy, limited stability, and high production costs hinder their clinical translation. Despite advances, most strategies still focus on either antiadhesive or antibacterial activity alone, with limited ability to regulate surface states that govern both microbial adhesion and host compatibility.^25^

Peptoids, *N*-substituted glycine oligomers, have emerged as promising alternatives to AMPs owing to their inherent proteolytic stability, a consequence of abiotic backbone structure.^26–29^ Solid-phase submonomer synthesis using diverse amine synthons enables sequence-defined incorporation of functional side chains and tunable self-assembly via noncovalent interactions.^30^ These features allow peptoids to recapitulate the structures and functions of diverse proteins such as antifreeze proteins, AMPs, and antibodies, making them attractive for biomedical applications.^31–34^ In particular, antimicrobial peptoids (ampetoids) emulate the amphipathic helical conformation of AMPs and exhibit potent antimicrobial activity against MDR pathogens through membrane disruption and intracellular targeting, while maintaining low cytotoxicity and exceptional proteolytic stability. Barron and co-workers further demonstrated the strong antibiofilm efficacy of self-assembling ampetoids against MDR strains, underscoring their potential as next-generation antibiofilm materials.^35,36^ Despite such promise, peptoids may exhibit unpredictable behaviors due to self-assembly–driven aggregation. Regulating their supramolecular organization is therefore critical to balancing structure and function, enabling the rational design of multifunctional materials.^35^

Here, we present a supramolecular design strategy for antibiofilm hydrogels by incorporating thiol-functionalized ampetoids into a norbornene-modified gelatin matrix via thiol–ene click chemistry. By precisely tuning the thiol-to-norbornene stoichiometric ratio, we established stoichiometric supramolecular control over ampetoid assembly states within the hydrogel network. This approach enabled defined transitions between disassembled and aggregated regimes, where the assembly state, rather than the mere change in antimicrobial dosage, reprogrammed the interfacial surface state of the hydrogel. This strategy translated molecular organization into tunable macroscopic properties, including surface hydrophilicity, antiadhesive behavior, bactericidal activity, and cytocompatibility. The optimized hydrogel, featuring fully cross-linked ampetoids, exhibited a synergistic balance of antiadhesive and antimicrobial functions while maintaining minimal cytotoxicity. Consistent with these attributes, it effectively suppressed biofilm formation by *S. aureus* and *P. aeruginosa*. Furthermore, the hydrogel was conformally applied to diverse substrates using scalable techniques such as doctor-blading and spray-coating, highlighting its applicability in contamination-prone, dynamically deforming medical environments. This work establishes a versatile platform for advanced antibiofilm surface coatings, enabled by rational engineering of peptoid–gelatin nanostructures.

To develop hydrogels integrated with ampetoids for effective biofilm inhibition, we rationally engineered their molecular sequence for optimal incorporation into a hydrogel network. Our design employed a 12-mer ampetoid composed of a repeating motif of two *N*-(S)-phenylethylglycine (*N*spe) and one *N*-(4-aminobutyl)­glycine (*N*Lys) residue (Figure S1). This amphiphilic structure typically folds into a polyproline type I-like helix conformation.^34^ These helices spontaneously self-assemble into micelles or bundled nanostructures. Such assemblies interact efficiently with bacterial membranes via electrostatic attraction, promoting membrane disruption and intracellular delivery.^35,37^ However, their cationic surfaces can paradoxically compromise the antiadhesive performance of hydrogels and induce cytotoxicity through undesirable interactions with mammalian cell membranes.^38,39^ To reconcile antibacterial activity with antiadhesion, we introduced *N*-(2-thioethyl)­glycine (*N*Cys) residues at both termini of the ampetoid sequence. This terminally modified ampetoid was designated TM1S. The terminal cross-linking approach was employed to suppress spontaneous self-assembly by covalently anchoring TM1S within the hydrogel matrix via thiol–ene click chemistry. Molecular dispersion of TM1S within the network was thus expected to minimize undesired multivalent interactions with bacterial surfaces while maintaining antibacterial activity. TM1S was successfully synthesized and characterized by mass spectrometry (MS) and analytical high-performance liquid chromatography (HPLC) (Figures S2 and S3). Gelatin was selected as the hydrogel matrix due to its intrinsic biocompatibility, low cost, and facile chemical modification using amine functionalities. Norbornene (Nb) moieties were introduced into gelatin through nucleophilic acyl substitution, enabling orthogonal cross-linking with the terminal *N*Cys of TM1S through thiol–ene chemistry. The degree of Nb substitution was quantified by proton nuclear magnetic resonance (^1^H NMR) using sodium 3-(trimethylsilyl)-1-propanesulfonate (DSS) as an internal standard, which revealed a substitution degree of 0.019 mmol Nb g^–1^ of gelatin (Figure S4).

Ampetoid-incorporated gelatin hydrogels were fabricated by incorporating TM1S cross-linkers into Nb-functionalized gelatin via thiol–ene photocoupling using lithium phenyl-2,4,6-trimethylbenzoylphosphinate (LAP) as the photoinitiator under UV irradiation at 365 nm (30 mW cm^–2^) for 10 min (Figure 1a). Cross-linking density was tuned by varying the molar ratio (*R*) of terminal *N*Cys groups to Nb moieties, yielding three formulations: Gel-1 (*R* = 1), Gel-2 (*R* = 2), and Gel-3 (*R* = 4). In Gel-1, all *N*Cys groups were stoichiometrically coupled with Nb moieties, achieving complete covalent immobilization of TM1S within the hydrogel network. In contrast, Gel-2 and Gel-3 contained excess TM1S, leaving a fraction of unreacted TM1S capable of self-assembly within the matrix. Successful gelation of all formulations was confirmed by inverted vial tests (Figure 1b). Oscillatory rheology revealed their viscoelastic behavior for all compositions, with the storage modulus (*G*′) consistently exceeding the loss modulus (*G*″) across the entire frequency range, indicating stable network formation (Figure 1c). Notably, increasing TM1S concentration led to a visible transition from transparent (Gel-1) to opaque (Gel-3), suggesting that unbound TM1S formed nanoscale assemblies within the hydrogel network. To elucidate the influence of TM1S concentration on supramolecular organization in both solution and hydrogel states, a series of structural analyses was conducted. We first examined the inherent self-assembly of TM1S in solution. Transmission electron microscopy (TEM) revealed concentration-dependent spherical nanostructures in phosphate-buffered saline (PBS) (Figure S5). At 7.7 mM (Gel-1), TM1S formed assemblies of approximately 12 nm, whereas at 30.8 mM (Gel-3), significantly larger assemblies of around 33 nm were observed. These results were corroborated by small-angle X-ray scattering (SAXS), which showed enhanced scattering intensity in the low-*q* region at elevated TM1S concentrations, consistent with increased supramolecular assembly (Figure S6). Notably, the organizational behavior of TM1S within the hydrogels differed markedly from that in solution. Scanning electron microscopy (SEM) revealed that Gel-Nb and Gel-1 exhibited porous networks without discernible nanostructures, likely due to complete covalent immobilization of TM1S within the hydrogel network (Figure 2a). In contrast, Gel-3 displayed densely distributed spherical assemblies (30–50 nm) within the network, indicating that excess, unbound TM1S retained its self-assembly tendency to form aggregates within the hydrogel matrix. These morphological observations were further supported by SAXS analysis of the hydrogels (Figure 2b). Gel-1 showed attenuated low-*q* scattering after UV cross-linking, suggesting a reduction in large-scale TM1S assemblies upon covalent tethering. However, Gel-3 retained a broad scattering profile characteristic of assembled structures, indicating the persistence of nanoscale assemblies due to its substantial content of unimmobilized TM1S.

**Figure 1:**
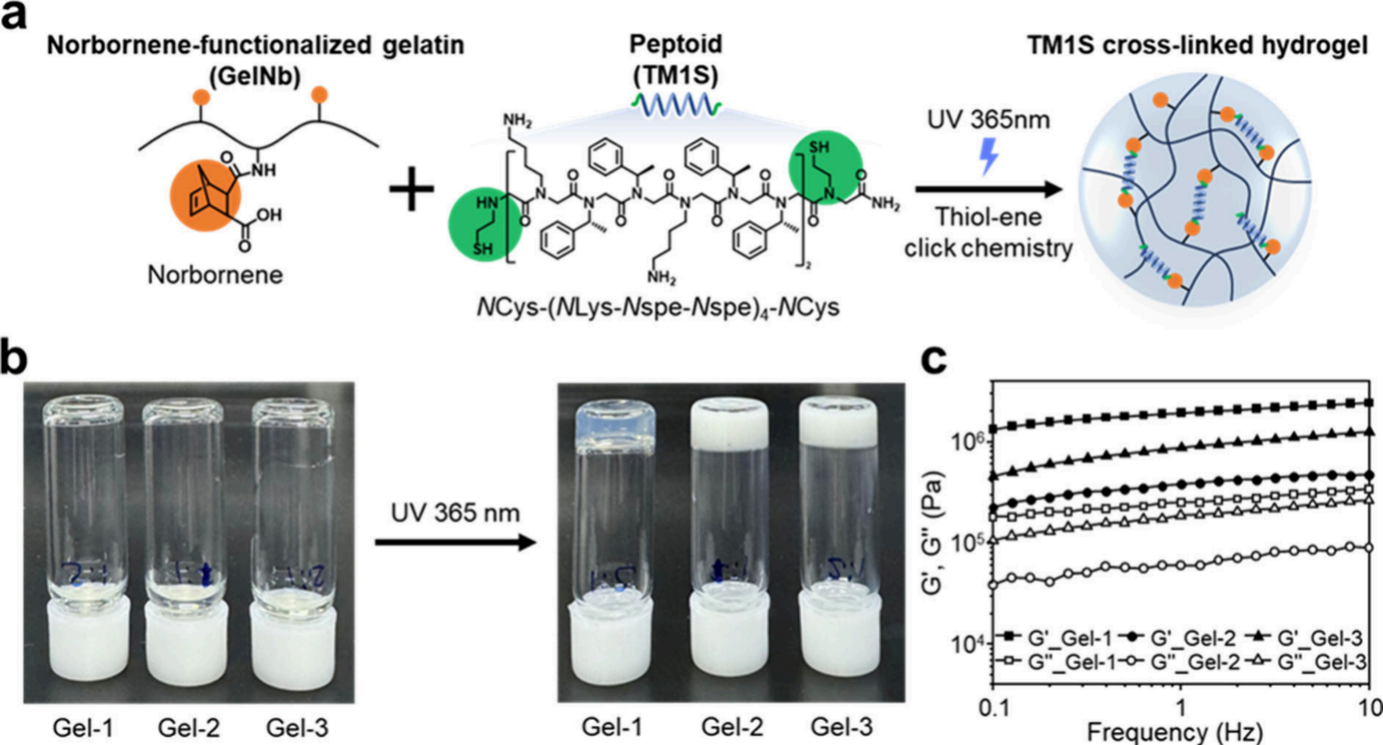
Design and synthesis of ampetoid-incorporated hydrogels. (a) Synthetic scheme of TM1S-incorporated hydrogels using TM1S as a photo-cross-linker for gelatin hydrogel. (b) Photographs of inverted vials of TM1S-incorporated hydrogels before (left) and after (right) gelation. (c) Frequency sweep analysis of TM1S-incorporated hydrogels measured from 0.1 to 10 Hz with a constant shear strain of 0.1%.

**Figure 2:**
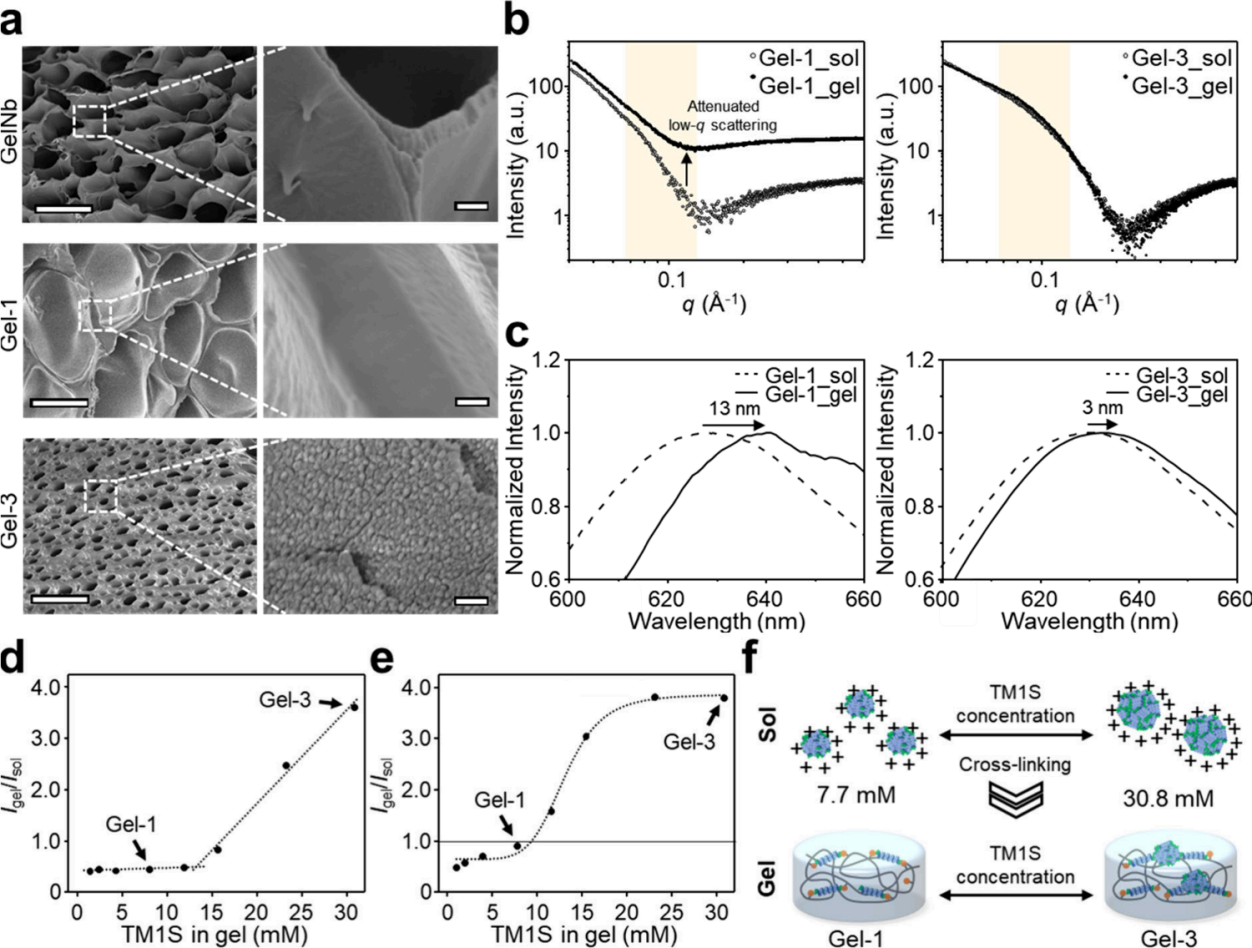
Structure analyses of TM1S-incorporated hydrogels. (a) SEM images of Nb-functionalized gelatin (Gel-Nb) hydrogel, Gel-1, and Gel-3. TM1S assemblies were visible in Gel-3. The scale bar represents 10 μm (left) and 200 nm (right). (b) SAXS profiles of Gel-1 and Gel-3 before and after gelation. Gel-1 showed a significant attenuation in the low-*q* scattering intensity upon gelation, indicating the disruption of pre-existing ampeptoid assemblies. In contrast, Gel-3 retained a broad scattering profile, suggesting the persistence of supramolecular assemblies within the hydrogel network. (c) Normalized fluorescence spectra of Nile Red in Gel-1 and Gel-3 before and after gelation. The spectra were compared based on relative peak shift. (d) Concentration-dependent change in Nile Red fluorescence upon gelation, shown as the relative maximum fluorescence intensity ratio (*I*_gel_/*I*_sol_) as a function of TM1S concentration. (e) Concentration-dependent change in pyrene fluorescence upon gelation, shown as the relative fluorescence intensity ratio (*I*_gel_/*I*_sol_) determined at 394 nm as a function of TM1S concentration. The dashed line represents a Hill-type sigmoidal fit (*R* ^2^ = 0.992, *n*_*H*_ = 6.3). (f) Schematic of proposed mechanism illustrating concentration-dependent changes in TM1S assembly during hydrogel cross-linking.

Further insight was obtained using the Nile Red assay, a fluorescence-based probe for hydrophobic environments (Figures 2c and S7).^40,41^ Following gelation, both Gel-1 and Gel-3 exhibited red-shifted emission and diminished fluorescence intensity relative to pregel solutions, indicating alterations in hydrophobic domains of TM1S assemblies. Gel-1 showed a 10 nm red-shift and an approximately 94% decrease in fluorescence intensity, reflecting significant loss of hydrophobic domain. Unlike Gel-1, Gel-3 exhibited only a 22% reduction in fluorescence intensity with minimal spectral shift, suggesting preservation of localized hydrophobic cores of TM1S assemblies. Analysis of the concentration-dependent change in Nile Red fluorescence revealed two distinct regimes: strong quenching at lower TM1S concentrations and an attenuated response at higher TM1S concentrations (Figure 2d). To independently validate these regime-dependent behaviors, we examined pyrene fluorescence across varying with TM1S concentrations before and after gelation (Figure S8). Pyrene fluorescence is highly sensitive to solvent exposure and molecular confinement,^42^ as quantified in Figure 2e, the ratio of pyrene fluorescence intensity after gelation (*I*_gel_/*I*_sol_) decreased at low TM1S concentrations, indicating that preformed TM1S assemblies were disrupted upon gelation. Conversely, a sharp increase of *I*_gel_/*I*_sol_ was observed beyond a threshold concentration (∼ 9.4 mM). Quantitative fitting of this response using a Hill-type sigmoidal function (*R* ^2^ = 0.992) showed excellent agreement with the experimental data, yielding a Hill coefficient (*n*_H_) of 6.3. This high cooperativity indicated a distinct, regime-dependent structural transition of TM1S assemblies within the hydrogel matrix.

Based on the combined structural and fluorescence analyses, we propose a model for the internal nanostructural organization of TM1S-incorporated gelatin hydrogels (Figure 2f). In aqueous solution, TM1S forms spherical amphiphilic assemblies. Upon gelation, the fate of these assemblies is governed by the stoichiometric balance between *N*Cys monomers of TM1S and norbornene groups on the gelatin backbone. In Gel-1, where TM1S is present at near-stoichiometric ratios relative to Nb, photoinitiated thiol–ene cross-linking leads to extensive covalent immobilization of TM1S within the gelatin network, disrupting the pre-existing supramolecular organization. This results in a loss of well-defined nanoscale assemblies and a transition toward a more molecularly dispersed TM1S distribution. This structural disintegration is consistent with attenuated low-*q* scattering and fluorescence quenching. In contrast, Gel-3 contains an excess of TM1S relative to Nb, allowing a significant fraction of TM1S to remain unbound after gelation. These unimmobilized TM1S remain supramolecularly associated rather than dispersed, yielding a mixed system of covalently tethered and supramolecularly assembled domains. This dual organization accounts for the persistence of nanoscale structures observed in microscopy and scattering experiments, as well as the cooperative fluorescence responses at higher TM1S concentrations.

We next evaluated the antiadhesive properties of TM1S-incorporated gelatin hydrogels. We hypothesized that in Gel-1, where TM1S is covalently immobilized and molecularly dispersed, multivalent interactions with bacterial surfaces would be minimized, thereby enhancing antiadhesive performance relative to Gel-3, which retains self-assembled domains with multicationic surfaces (Figure 3a). To test this, we quantified protein and bacterial adsorption using fluorescence emission measurements (Figure 3b and S9). In this assay, fluorescein-conjugated bovine serum albumin (FITC-BSA) served as a model protein because of its net negative charge at physiological conditions (pH 7.4).^43^ Consistent with our hypothesis, Gel-3 exhibited higher adsorption of both FITC-BSA, *E. coli, S. aureus, and P. aeruginosa* compared to Gel-1, indicating that the internal organization of TM1S directly influenced surface adhesion behavior. Surface zeta-potential measurements supported a clear distinction of adhesion behavior between the two hydrogels: Gel-3 exhibited a positively charged surface (4.73 mV), whereas Gel-1 showed a net negative surface potential (−6.03 mV) comparable to that of Gel-Nb, despite the cationic nature of TM1S in solution (Figure 3c and S10). We further conducted contact angle measurements to assess the hydrophilicity of the hydrogel surface (Figure 3d and S11). Incorporation of TM1S increased surface hydrophobicity compared to unmodified Gel-Nb hydrogel. Notably, Gel-1 exhibited a higher contact angle than Gel-3, despite its lower TM1S content. This trend was attributed to the molecularly dispersed state of TM1S in Gel-1, which maximizes the exposure of hydrophobic residues at the interface. In contrast, in Gel-3, TM1S underwent supramolecular assembly, which sequestered hydrophobic moieties within the interior cores while presenting cationic groups at the surface. These results suggest that the multivalent, localized cationic domains in Gel-3 facilitated strong electrostatic interactions with proteins and bacteria, thereby promoting adhesion (Figure S9), whereas covalently immobilized TM1S in Gel-1 minimized such interactions, leading to reduced adhesion.

**Figure 3:**
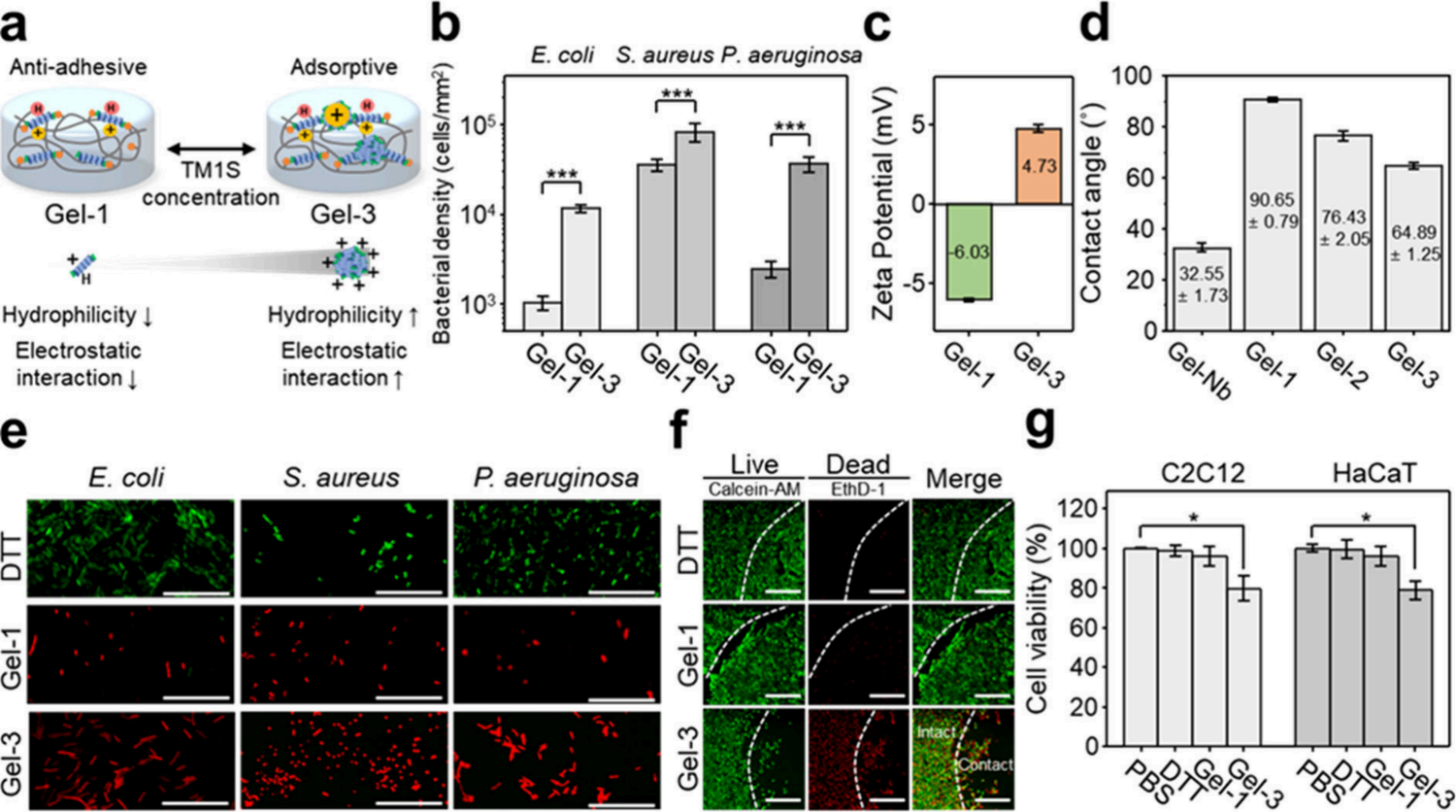
Antiadhesive and antimicrobial properties of TM1S-incorporated hydrogels. (a) Proposed scheme for antiadhesive and antimicrobial properties of TM1S-incorporated hydrogels. Concentration-dependent modulation of TM1S self-assembly, altering the surface presentation of cationic (+) and hydrophobic (H) moieties. (b) Quantification of adhered bacteria on hydrogel surfaces to assess antiadhesive performance, quantified as bacterial density (mean ± SD, *n* = 8). Statistical significance was assessed using Welch’s *t* test, with significance defined at *p* < 0.001 (***). (c) Surface zeta potentials of Gel-1 and Gel-3 measured by streaming potential (mean ± SD, *n* = 3). (d) Static contact angle measurements using 3 μL PBS droplets (mean ± SD, *n* = 3). (e) Antimicrobial activity evaluated by Live/Dead staining of bacteria on hydrogel surfaces. The scale bar represents 20 μm. (f) Live/Dead-stained images of C2C12 cells after incubation with TM1S-incorporated hydrogels to evaluate cytotoxicity. The scale bar represents 200 μm. (g) Quantitative cell viability of C2C12 myoblasts and HaCaT keratinocytes after exposure to PBS and hydrogel samples (DTT, Gel-1, and Gel-3), as determined by CCK-8 assay (mean ± SD, *n* = 3). Error bars represent standard deviation (S.D.). Statistical significance was assessed using Welch’s *t* test, with significance defined at *p* < 0.05 (*).

The antibacterial activity of TM1S-incorporated hydrogels was evaluated against *E. coli* (ATCC25922), a Gram-negative strain widely used for antimicrobial activity evaluations. We further extended these evaluations to include *S. aureus* (ATCC25923), and *P. aeruginosa* (PAO1), representing both Gram-negative and Gram-positive clinically relevant pathogens. Live/Dead fluorescence imaging revealed strong bactericidal activity across all TM1S-incorporated hydrogels, regardless of concentration or structural configuration (Figure 3e and S12). In contrast, DTT-cross-linked gelatin hydrogels, used as thermally stable negative controls, exhibited no antibacterial effect, confirming that the antibacterial activity originated from TM1S. Consistent with these observations, minimum inhibitory concentration (MIC) and minimum bactericidal concentration (MBC) assays supported that TM1S exerted its antibacterial effect through bacterial membrane disruption (Table S1). Notably, eluates collected from sufficiently washed hydrogels exhibited no detectable antibacterial activity against *E. coli* (Figure S13). In addition, *E. coli* present in the supernatant remained viable after incubation with TM1S-incorporated gelatin hydrogels (Figure S14). This finding suggests that the antibacterial effect is predominantly mediated by contact-killing at the hydrogel interface rather than the release of soluble antibacterial species.

Cytotoxicity of TM1S-incorporated gelatin hydrogels was assessed at the hydrogel–cell interface by coincubating C2C12 myoblasts and HaCaT keratinocytes, followed by Live/Dead staining.^44^ For both cell types, cells in contact with Gel-1 displayed strong green fluorescence, indicating high viability with negligible cytotoxicity, whereas Gel-3 induced pronounced red fluorescence as a dead-cell signal (Figure 3f, S15, and S16). To complement these qualitative observations, quantitative cell viability was further evaluated using a CCK-8 metabolic assay. Consistent with the Live/Dead imaging results, Gel-3 reduced viability to approximately 80% in both C2C12 myoblasts and HaCaT keratinocytes, indicating evident cytotoxicity (Figure 3g). Remarkably, Gel-1 exhibited no detectable cytotoxicity, even at peptoid concentrations orders of magnitude higher than the half-maximal lethal concentration (LC_50_) reported for the parent peptoid (8 μM against MRC5 fibroblasts, MTS assay), although structural differences between TM1S and the parent analogue limit direct quantitative comparison.^45^ Since TM1S concentration was the only varying parameter among formulations, the observed differences in cytotoxicity could be attributed to the structural configuration of TM1S. In Gel-1, covalent immobilization of TM1S suppressed multivalent cationic domains, thereby diminishing electrostatic interactions with mammalian membranes. Washing experiments further confirmed minimal release of TM1S from the hydrogel matrix (Figure S14), at concentrations insufficient to induce cytotoxic effects. Thus, the distinct cytotoxic behaviors of Gel-1 and Gel-3 primarily arise from differences in supramolecular organization rather than TM1S dosage or release.

Collectively, precise tuning of cross-linker stoichiometry within the hydrogel matrix modulated peptoid self-assembly and enabled nanoscale engineering of hydrogel microenvironments, which in turn strongly influenced surface charge distribution and cell–material interactions. A spectrum of structural states, ranging from well-defined nanostructures to fully disassembled ampetoids, produced distinct properties in antiadhesive behavior and cytocompatibility. Gel-1, where TM1S was covalently immobilized and disassembled within the matrix, exhibited a balanced biological profile, with reduced protein and bacterial attachment and minimal cytotoxicity toward C2C12 myoblasts. In contrast, Gel-3, containing highly assembled TM1S nanostructures, displayed strong protein and bacterial adhesion together with pronounced cytotoxicity, both attributable to abundant multivalent cationic domains. These adverse effects highlight how excessive supramolecular cohesion compromises hydrogel biocompatibility. Overall, our findings demonstrated that the regulation of TM1S assembly was a key determinant of hydrogel multifunctionality, extending beyond antimicrobial potency.

Building on these findings, we investigated the antibiofilm efficacy of TM1S-incorporated hydrogels. Gel-1, selected for its balanced antibacterial, antiadhesive, and biocompatible properties, was quantitatively assessed against *S. aureus* and *P. aeruginosa*. Biofilm biomass was quantified by crystal violet staining after incubation in the presence or absence of Gel-1 (Figure 4a). Treatment with Gel-1 markedly suppressed biofilm formation, reducing biomass to 40.8% for *S. aureus* and 42.0% for *P. aeruginosa* relative to untreated controls. This suppression was visually evident as a color change from deep to pale violet. 3D confocal laser scanning microscopy (CLSM)-based live/dead imaging revealed an increase in membrane-compromised bacteria within Gel-1-treated biofilms compared to untreated controls (Figure S18). However, quantitative analysis indicated that bacterial killing alone did not fully account for the total reduction in biomass, suggesting that factors beyond direct bactericidal activity should contribute to the biofilm inhibition. We therefore examined changes in the extracellular polymeric substance (EPS) matrix and three-dimensional biofilm architecture by CLSM. While untreated samples exhibited dense and continuous EPS networks with minimal dead signals, Gel-1 treatment resulted in significant disruption and loss of EPS continuity (Figure 4b). Consistent with these observations, orthogonal (x–z) sections revealed a substantial collapse in the vertical organization of the EPS matrix in Gel-1 treatment (Figure 4c). Quantitative analysis further confirmed a marked reduction in biofilm thickness compared to untreated controls (Figure 4d). Upon Gel-1 treatment, thickness of *S. aureus* and *P. aeruginosa* biofilms decreased from 1.18 ± 0.60 and 3.93 ± 1.11 μm to 0.22 ± 0.19 and 1.84 ± 0.97 μm, respectively. Scanning electron microscopy (SEM) provided complementary morphological evidence (Figure 4e). While untreated samples exhibited mature biofilms with dense microcolonial structures and a continuous EPS-rich matrix, sparse bacterial coverage, localized membrane deformation and structural damage occurred under Gel-1 treatment. Based on these results, we propose a synergistic mechanistic model for the antibiofilm activity of Gel-1. The molecularly dispersed state of TM1S in Gel-1 endows the hydrogel surface with enhanced hydrophobicity, which imposes a sustained interfacial stress on arriving bacteria. This hydrophobic environment effectively hinders initial bacterial docking and disrupts the subsequent accumulation of EPS. Concurrently, any adhered bacteria are neutralized via a contact-killing mechanism. This synergistic effect prevents the transition from initial attachment to the formation of a mature three-dimensional biofilm.

**Figure 4:**
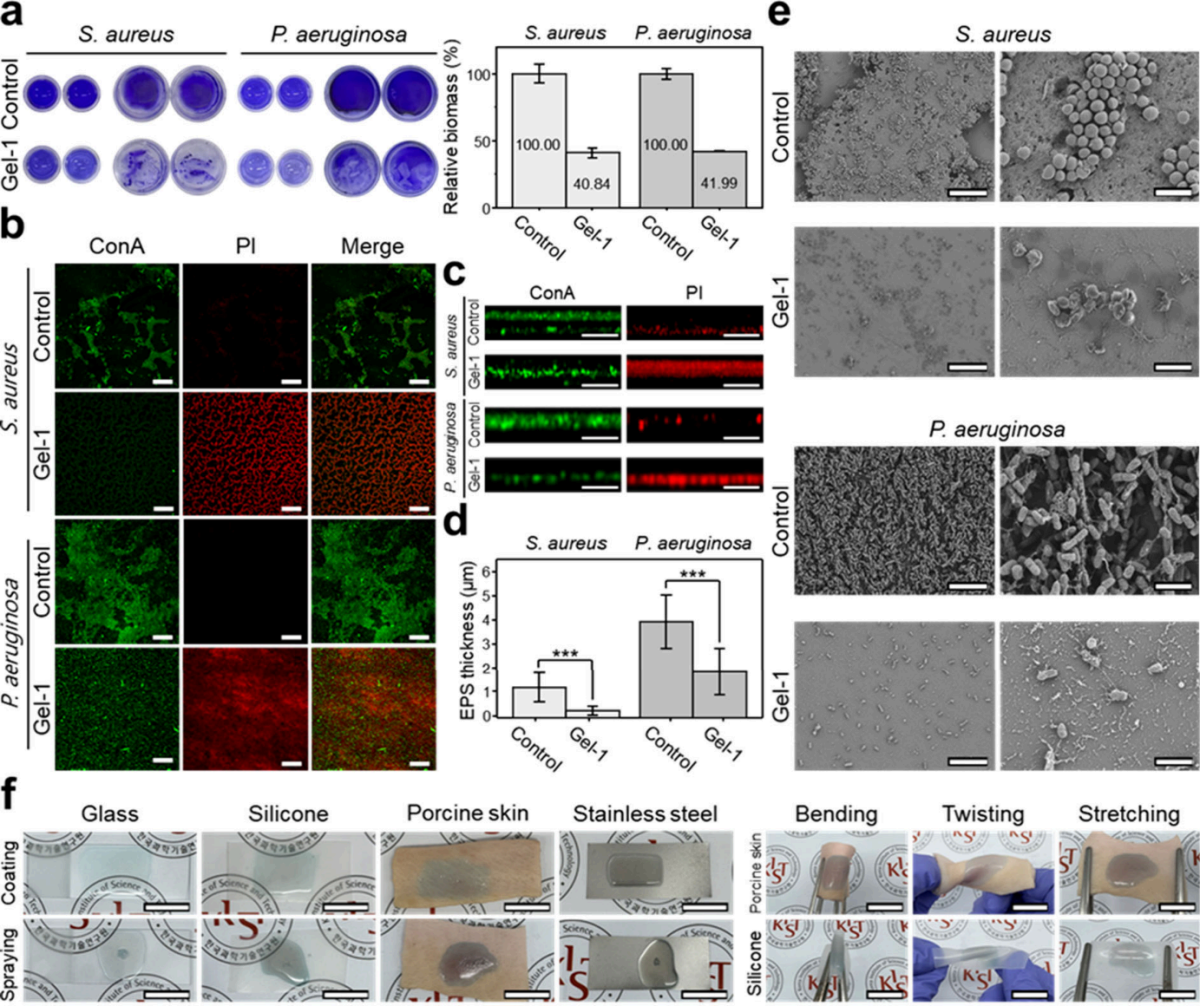
Biofilm inhibition by TM1S-incorporated hydrogels. (a) Quantification of biofilm biomass on TM1S-incorporated hydrogels via crystal violet staining (mean ± SD, *n* = 3). (b) CLSM images of biofilms stained for EPS matrix and dead bacteria after Gel-1 treatment. ConA (green) labels the EPS matrix and PI (red) labels membrane-compromised bacteria. Z-stacked projections are shown. Scale bars represent 50 μm. (c) Orthogonal (x–z) CLSM images extracted from the same z-stack data sets, highlighting the vertical architecture and thickness of the biofilms. Scale bars represent 10 μm. (d) Quantification of biofilm thickness derived from CLSM z-stack analysis. Biofilm thickness was calculated based on the ConA-labeled EPS matrix (mean ± SD, *n* > 30). Statistical significance was assessed using Welch’s *t* test, with significance defined at *p* < 0.001 (***). (e) SEM images of biofilms treated with TM1S-incorporated hydrogels. The scale bar represents 10 μm (left) and 2 μm (right). (f) Hydrogels applied to glass, silicone, porcine skin, and stainless steel using coating and spray methods. No cracks or detachments were observed at interfaces during bending, twisting, or stretching tests. The scale bar represents 20 mm.

Lastly, we evaluated the practical applicability of Gel-1 hydrogels by coating biomedically relevant substrates using doctor-blading and spray-coating techniques (Figure 4f). The hydrogels exhibited excellent flexibility and conformability on glass, porcine skin, silicone, and stainless-steel surfaces under both static and dynamically deformed conditions. Notably, the coatings remained intact on silicone and porcine skin during stretching, bending, and twisting, demonstrating strong interfacial adhesion and mechanical robustness. To further assess mechanical stability under physiological conditions, the swelling and mechanical properties of Gel-1 were characterized in PBS over an extended period. First, swelling kinetic analysis revealed that Gel-1 rapidly reached a swelling plateau within 2 h and subsequently maintained a stable hydration state thereafter (Figure S19a). Following this equilibration, the mechanical integrity of the hydrogel was evaluated. Compressive stress–strain curves remained nearly unchanged even after up to 5 days of swelling in PBS, indicating robust hydrolytic and structural stability (Figure S19b). Moreover, Gel-1 exhibited large, fully recoverable deformation with minimal hysteresis at compressive strains up to 70% (Figure S19c) and showed negligible mechanical degradation over repeated loading–unloading cycles (Figure S19d). Collectively, these results supported the suitability of Gel-1 hydrogel coatings for long-term integration with dynamic biological interfaces.

In this study, we developed a multifunctional hydrogel platform incorporating ampetoids that integrates antiadhesive, bactericidal, and cytocompatible functions to inhibit biofilm formation. Our approach leveraged thiol–ene click chemistry to precisely control the nanoscale organization of TM1S peptoid cross-linkers within a gelatin matrix by modulating *N*Cys/Nb molar ratios. This molecular-level regulation translated into macroscopic control of hydrogel properties, including surface hydrophilicity, protein and bacterial adhesion, and cytocompatibility. Gel-1, characterized by fully immobilized and disassembled TM1S, demonstrated the most balanced biological attributes, combining antifouling capacity, potent contact-dependent bactericidal effects, and negligible cytotoxicity toward C2C12 myoblasts. Therefore, Gel-1 showed markedly inhibited biofilm formation by both *S. aureus* and *P. aeruginosa*. Its practical applicability was further demonstrated by conformal, mechanically robust coatings on diverse medically relevant substrates via doctor-blading or spray-coating, maintaining structural integrity under dynamic deformation. Overall, this work establishes a tunable supramolecular design strategy for engineering peptoid nanostructures within hydrogels, offering broad potential for infection-resistant medical device coatings, advanced wound dressings, and next-generation biointerfaces to address the urgent challenge of antimicrobial resistance.

## Supplementary Material



## Notes

Materials; synthesis methods of TM1S, Nb-functionalized gelatin (Gel-Nb), and TM1S-incorporated hydrogels; characterizations of TM1S assemblies; characterizations of nanostructures, antimicrobial properties, antiadhesive properties, and antibiofilm properties of TM1S-incorporated hydrogels; submonomers and structure of TM1S; calculation of Nb functional groups of Gel-Nb; TEM images and SAXS spectra of TM1S; protein-adsorption, contact angle, and cytotoxicity tests of TM1S-incorporated hydrogels; zeta potential analysis and antibacterial test of hydrogel components (PDF)
