# Supplementary material for: Modulation of Peptoid Nanostructure for Antibiofilm Hydrogel Interfaces
Source: Nano Lett. 2026 Feb 26;26(9):3003–11. doi: 10.1021/acs.nanolett.5c05266 (PMC12983360; doi:10.1021/acs.nanolett.5c05266)
Supplement: Supplementary file 1 [file nl5c05266_si_001.pdf]

Supporting Information

Modulation of Peptoid Nanostructure for Antibiofilm Hydrogel Interfaces

Jae Won Yun[1,5]†, Il-Soo Park[2]†, Heewoong Yoon[3]†, Jiwon Woo[4], Dong-Yeong Kim[2], Woojin Yang[2], Jieun Choi[3], Dal-Hee Min[4], Jung-Hyun Lee[5], Jiwon Seo[3], Jae Hong Kim[1,2,6]**

[1] Convergence Research Center for Solutions to Electromagnetic Interference in Future-mobility, Korea Institute of Science and Technology (KIST), Seoul 02792, Republic of Korea

[2] Electronic and Hybrid Materials Research Center, Korea Institute of Science and Technology (KIST), Seoul 02792, Republic of Korea

[3] Department of Chemistry, Gwangju Institute of Science and Technology (GIST), Gwangju 61005, Republic of Korea

[4] Department of Chemistry, Seoul National University, Seoul, 08826, Republic of Korea

[5] Department of Chemical and Biological Engineering, Korea University, Seoul 02841, Republic of Korea

[6] GIST InnoCORE AI-Nano Convergence Institute for Early Detection of Neurodegenerative Diseases, Gwangju Institute of Science and Technology, Gwangju, Republic of Korea

† These authors contributed equally.

*E-mail: jaehongkim@kist.re.kr and jseo@gist.ac.kr

Table of contents

Materials and Methods 3

Figure S1. Chemical structures of peptoid monomers and TM1S 9

Figure S2. MALDI-TOF mass spectrum of TM1S 10

Figure S3. HPLC chromatogram of TM1S 11

Figure S4. Synthesis scheme and Nb functionalization of Gel-Nb 12

Figure S5. TEM images and size distribution of TM1S assemblies in PBS 13

Figure S6. SAXS spectra of TM1S assemblies at varying concentrations 14

Figure S7. Nile Red fluorescence response to gelation in TM1S-containing systems 15

Figure S8. Pyrene fluorescence spectra of TM1S-containing systems 16

Figure S9. FITC-BSA adsorption on TM1S-incorporated hydrogel surfaces 17

Figure S10. Zeta potentials of TM1S-incorporated hydrogel components 18

Figure S11. Contact angle images of TM1S-incorporated hydrogels 19

Figure S12. Individual fluorescence channels for Live/Dead imaging (Figure 3e) 20

Table S1. MIC and MBC values of hydrogel components against bacteria 21

Figure S13. Validation of TM1S immobilization in TM1S-incorporated hydrogels 22

Figure S14. Live/dead imaging of *E. coli* in hydrogel supernatants 23

Figure S15. Cytotoxicity of Gel-3 in C2C12 cells 24

Figure S16. Cytotoxicity of TM1S-incorporated hydrogels in HaCaT cells 25

Figure S17. Relative TM1S release from TM1S-incorporated hydrogels 26

Figure S18. Viability analysis of *S. aureus* and *P. aeruginosa* biofilms on Gel-1 27

Figure S19. Mechanical stability and deformation behavior of Gel-1 28

References 29

Materials and methods

Materials. All chemicals and solvents were obtained from commercial suppliers and used without further purification. The Fmoc-Rink Amide MBHA resin (100–200 mesh, 0.65 mmol/g) was purchased from Merck Millipore (Billerica, MA, USA). Acetonitrile (ACN, HPLC grade) was obtained from Avantor (Radnor, PA, USA). Trityl chloride (97%), cysteamine hydrochloride (≥98%), triisopropylsilane (TIS, 98%), phosphate-buffered saline (PBS, pH 7.4), Mueller–Hinton Broth II (MHB2, cation-adjusted), *N*-(S)-phenylethylglycine (*N*spe, 98%), gelatin from bovine skin (Type B), lithium phenyl-2,4,6-trimethylbenzoylphosphinate (LAP, ≥95%), dithiothreitol (DTT, 97%), pyrene (98%), fluorescein-conjugated bovine serum albumin (FITC-BSA), and crystal violet (≥90%) were purchased from Sigma-Aldrich (St. Louis, MO, USA). *N,N*-Dimethylformamide (DMF, 99.8%), 1-methyl-2-pyrrolidinone (NMP, 99.5%), trifluoroacetic acid (TFA, 99.5%), SYTO 9 and propidium iodide (LIVE/DEAD™ BacLight™ bacterial viability kit), calcein-AM and ethidium homodimer-1 (EthD-1) (LIVE/DEAD™ Viability/Cytotoxicity Kit for mammalian cells), concanavalin A-Alexa Fluor 488 conjugate, Nile Red (99%), and Dulbecco's Modified Eagle Medium (DMEM) were purchased from Thermo Fisher Scientific (Waltham, MA, USA). Cell Counting Kit-8 (CCK-8) was purchased from Dojindo Molecular Technologies (Rockville, MD, USA). 1,4-Diaminobutane (>98.0%), di-tert-butyl dicarbonate (>95.0%), *N,N'*-diisopropylcarbodiimide (DIC, >98.0%), *N,N*-diisopropylethylamine (DIEA, >99.0%), 5-norbornene-2,3-dicarboxylic anhydride (>97.0%), and sodium 3-(trimethylsilyl)-1-propanesulfonate (>98.0%) were obtained from TCI (Tokyo, Japan). Milli-Q water was used for all HPLC purification and sample preparation steps.

Synthesis of TM1S. The peptoid was synthesized using the submonomer solid-phase synthesis protocol[1,2]. Submonomers *N*-(4-aminobutyl)glycine (*N*Lys) and *N*-(2-thioethyl)glycine (*N*Cys) were prepared following established procedures[3,4]. Peptoid synthesis was carried out in a solid-phase extraction cartridge equipped with a polyethylene filter (Applied Separations, USA) and agitated in a shaker incubator (LK LAB Korea, Republic of Korea). For each batch, 0.5 mmol (0.77 g) of Fmoc-Rink Amide MBHA resin was used. After each reaction step (Fmoc deprotection, bromoacetylation, and amine displacement), the resin was washed twice with DCM and twice with DMF. Fmoc deprotection was performed with 20% (v/v) piperidine in DMF, applied twice (5 and 15 min, 10 mL each) at room temperature. Bromoacetylation was conducted using a solution of bromoacetic acid (1.2 M in DMF, 20 equiv.) and DIC (20 equiv.) for 20 min at room temperature. Amine displacement was achieved by treating the resin with *N*Cys, *N*Lys, or *N*spe (1.0 M in NMP) at 37 °C for 30 min. These steps were repeated until the desired sequence was obtained. Peptoid cleavage from the resin was achieved using a TFA/triisopropylsilane/water solution (95:2.5:2.5, v/v/v) at room temperature for 30 min. The crude product was purified by preparative HPLC on a (Waters 2535 quaternary gradient module, 2489 UV/Visible detector, Fraction Collector III; Waters, USA) using a SunFire® Prep C18 OBD™ column (5 µm, 19 mm × 150 mm; Waters, USA). The mobile phase consisted of water and acetonitrile containing 0.1% TFA, with a linear gradient from 15% to 60% acetonitrile over 30 min at a flow rate of 10 mL/min, monitored at 220 nm. Product purity was confirmed by an analytical HPLC (Waters 1525 binary pump, 2489 UV/Visible detector, 2707 autosampler, column oven; Waters, USA) using a SunFire® C18 column (4.6 × 250 mm, 5 µm; Waters, USA). The column was equilibrated with 10% acetonitrile at 40 °C for 10 min. After injection, the condition was maintained for 5 min, followed by a linear gradient from 10% to 100% acetonitrile over 30 min at a flow rate of 1 mL/min, with detection at 220 nm.

Preparation of norbornene (Nb)-functionalized gelatin (Gel-Nb). Norbornene (Nb)-functionalized gelatin (Gel-Nb) was synthesized following established protocols[5,6]. A 10% (w/v) gelatin solution was prepared by dissolving gelatin in PBS at 50 °C. A 20% (w/v) 5-Norbornene-2,3-dicarboxylic anhydride solution was then added, and the pH was adjusted to 7.4 using 10 M NaOH. The mixture was stirred at 50 °C for 3 h and centrifuged at 3500 × g for 9 min to remove undissolved 5-norbornene-2,3-dicarboxylic anhydride. The supernatant was dialyzed against deionized water at 40 °C for 7 days (MWCO: 6–8 kDa) and lyophilized to yield a dry product. The product was sterilized under 254 nm UV light for 30 min and stored at -20 °C. The degree of norbornene functionalization (Nb_{mole}) was determined by ^1H NMR spectroscopy using sodium 3-(trimethylsilyl)-1-propanesulfonate (DSS) as an internal standard[7]. Briefly, 20 mg of Gel-Nb was dissolved in 1 mL D_2O containing 0.0573 mmol DSS per gram of gelatin. Nb_{mole} was calculated according to eq. (1):

$$\mathrm{Nb_{mole}} = \frac{I_{Nb}}{I_{DSS}} \times \frac{9\mathrm{H}}{2\mathrm{H}} \times \frac{0.0573\ \mathrm{mmol\ DSS}}{\mathrm{g\ gelatin}} \qquad \cdots(1)$$

Preparation of TM1S-incorporated gelatin hydrogels. All hydrogel precursors were individually dissolved in PBS prior to gelation. The Gel-Nb solution was preheated to 40 °C to ensure complete dissolution. Hydrogels were synthesized using 8% (w/v) Gel-Nb with 4 mM lithium phenyl-2,4,6-trimethylbenzoylphosphinate (LAP) as a photoinitiator. TM1S and dithiothreitol (DTT) were employed as cross-linkers at varying stoichiometric ratios of *N*Cys to Nb as R (R = 1, 2, or 4). For antimicrobial and antiadhesion assays, 200 µL of the pregel solutions were cast into disk-shaped hydrogels (10 mm in diameter). Cross-linking was induced by UV irradiation at 365 nm (30 mW/cm^2, total dose: 18,000 mJ/cm^2) for 10 min. The resulting hydrogels were washed with PBS at 37 °C by performing three cycles of 12 h using 20 mL of PBS per hydrogel disk prior to all subsequent experiments. For visualization, 200 µL of pregel solution containing 2 µM of brilliant blue was applied via doctor-blading or atomizer spraying.

Characterizations. Mass spectra were acquired by MALDI-TOF mass spectrometry on a Bruker Ultraflex III (Bruker, Germany) in positive ion mode. ^1H NMR spectra were recorded on a Bruker Avance III 400 MHz NMR spectrometer (Bruker, Germany) in D_2O. Hydrogel morphology and biofilm formation on coverslips were examined by scanning electron microscopy (SEM) on a Sigma 300 (ZEISS, Germany) operated at 2 kV accelerating voltage. Images of TM1S assemblies were obtained by energy-filtering transmission electron microscope (EF-TEM) on a LIBRA 120 (ZEISS, Germany) operated at 120 kV accelerating voltage. Live/Dead-stained bacteria on hydrogel surfaces and 3D biofilm images on coverslips were acquired by confocal laser scanning microscopy (CLSM) on an FV3000RS (Olympus, Japan). Critical point drying was perfored on EM CPD300 (CPD; Leica, Germany).

Rheological properties. Rheological measurements were performed on an MCR302e rheometer (Anton Paar, Austria). A 200 µL sample of TM1S-incorporated hydrogel was loaded onto the stage with an 8 mm parallel plate geometry and a 1 mm gap. Storage modulus (G') and loss modulus (G") were recorded under a frequency sweep (0.1–10 Hz) at a constant shear strain of 0.1%. All measurements were conducted at 40 °C.

Small-angle X-ray scattering (SAXS). SAXS measurements were conducted at the 4C beamline of PLS-II (Pohang Accelerator Laboratory, Republic of Korea)[8]. A synchrotron X-

ray source (16.9 keV, beam size 100 μm × 30 μm) and a Rayonix SX165 CCD detector (Rayonix, USA) were used. Data were collected at a sample-to-detector distance of 1 m (q range: 0.04–0.6 Å^{-1}) with an exposure time of 30 s and processed using FIT2D software (ESRF, France). Samples were sealed in quartz capillaries (Hilgenberg, Germany) and analyzed at room temperature. Absolute intensity calibration was performed using PBS (for TM1S solutions) and 8% (w/v) Gel-Nb solution (for TM1S-incorporated hydrogels) as primary standards.

Fluorescence probe assays. Pregel solutions (500 μL) containing varying concentrations of TM1S were prepared and mixed with either Nile Red or pyrene from DMSO stock solutions. Nile Red was added to a final concentration of 2 μM from a 60 μM stock, while pyrene was added to a final concentration of 5 μM from a 60 μM stock. The mixtures were incubated at 37 °C under constant stirring for 1 h and subsequently dispensed into a 96-well plate (100 μL per well). Fluorescence emission spectra were recorded before and after cross-linking using a Synergy HTX multi-mode microplate reader (BioTek, USA). For Nile Red measurements, samples were excited at 510 nm and emission was collected from 550 to 700 nm. For pyrene measurements, excitation was set to 335 nm and emission was collected from 360 to 600 nm. Changes in fluorescence intensity and spectral features before and after gelation were analyzed to assess probe partitioning and confinement within TM1S-derived microenvironments.

Protein adsorption assay. Pregel solutions with varying TM1S concentrations were photo-crosslinked in 24-well plates (100 μL per well). The resulting hydrogels were incubated in PBS containing 0.02 mg/mL FITC-BSA (100 μL per well) at 37 °C for 2 h to allow protein adsorption on hydrogel surfaces. Unbound proteins were removed by washing three times with PBS. Fluorescence intensity was then measured using a Synergy HTX multi-mode microplate reader (BioTek, USA).

Zeta potential. Solution zeta potentials were measured using a Zetasizer Nano ZS (Malvern Instruments, UK) at a concentration of 0.1 wt% in 10 mM KCl and 1× PBS buffer, both at pH 7.4 (n = 3). Surface zeta potentials of hydrogel surfaces were determined by streaming potential measurements using a SurPASS electrokinetic analyzer (Anton Paar GmbH, Austria), following a previously reported procedure[9]. Measurements were carried out in 10 mM KCl solution at pH 7.4 (n = 3).

Contact angle. Pregel solutions with varying TM1S concentrations were blade-coated on silicon wafer and subsequently UV-crosslinked. Samples were mounted on the sample stage aligned with the light source, and a 3 μL droplet of PBS was deposited on each surface. Photographs were taken 180 s after deposition to ensure droplet stabilization. Multiple locations per sample were measured. Water contact angles were determined using the sessile drop method and analyzed in ImageJ software with a five-point fitting approach.

Antimicrobial assay. The antimicrobial efficacy of TM1S-incorporated hydrogels against *E. coli* (ATCC 25922), *S. aureus* (ATCC 25923), and *P. aeruginosa* (PAO1) was assessed by Live/Dead staining. Primary bacterial cultures were grown overnight in cation-adjusted MHB2 medium at 37 °C in a shaker incubator. Secondary cultures were prepared the following day, incubated for 3 h, and diluted in MHB2 for further use. Circular wells (8 mm diameter, 1 mm depth) were created using punched foam tape on glass slides. Pregel solution (90 μL) was introduced into each well and UV-crosslinked. Hydrogels were immersed in sterile PBS at

37 °C for three 12 h washing cycles to remove unreacted components, then air-dried. Bacterial suspensions (100 μL, 10^7 CFU/mL) were added to each well and incubated at 37 °C for 3 or 6 h. Samples were gently rinsed with PBS and stained with SYTO 9 and propidium iodide (PI) in the dark. Confocal images of bacteria on hydrogel surfaces were acquired by CLSM (λ_{ex} = 488 nm for SYTO 9; λ_{ex} = 561 nm for PI). For quantification, 50 images (80 μm × 80 μm) per sample were analyzed using ImageJ software. Using the same Live/Dead staining and CLSM conditions, bacterial viability in the supernatant after 6 h incubation with the hydrogels was additionally evaluated. Antibacterial activity of hydrogel eluates was further assessed by treating bacteria with eluates collected after hydrogel incubation, followed by Live/Dead staining under identical imaging conditions.

The antimicrobial activities of individual hydrogel components were further evaluated against *E. coli* (ATCC 25922), *S. aureus* (ATCC 25923), and *P. aeruginosa* (PAO1) using minimum inhibitory concentration (MIC) and minimum bactericidal concentration (MBC) assays. Hydrogel components were serially diluted in PBS (1.953 × 10^{-6} to 1 × 10^{-3} M) in a 96-well polypropylene plate. Bacterial suspension (100 μL, 2–5 × 10^5 CFU/mL) was added to each well and incubated at 37 °C for 24 h. Bacterial growth was monitored by measuring OD_{600} on a microplate reader. MIC was defined as the lowest concentration with no visible bacterial growth. For MBC determination, 100 μL of suspension from the MIC wells was streaked onto agar plates and incubated for 24 h at 37 °C. MBC was defined as the lowest concentration showing no visible bacterial colonies.

Cytotoxicity assay. Hydrogel cytotoxicity was evaluated against the C2C12 myoblasts (ATCC, USA) and HaCaT human keratinocytes (ATCC, USA) using Live/Dead staining and a CCK-8 metabolic assay. Hydrogel disks (200 μL) were pre-washed with PBS and incubated with 400 μL of fresh DMEM for 24 h in a 24-well plate. Meanwhile, C2C12 and HaCaT cells (1 × 10^5 cells per well) were seeded in a 12-well plate and cultured overnight under standard conditions (37 °C, 5% CO_2). After incubation, hydrogel disks were transferred to the cell-seeded wells and co-incubated for 4 h under the same conditions. The hydrogels and media were then removed, and the cells were stained with calcein-AM and ethidium homodimer-1 (EthD-1) for 30 min at 37 °C, followed by rinsing with PBS. The Live/Dead images were obtained on an IX71 fluorescence microscopy (Olympus, Japan). For quantitative analysis, absorbance values from the CCK-8 assay were measured at 450 nm with a reference wavelength of 670 nm using a Synergy HTX multi-mode microplate reader (BioTek, USA). Cell viability was calculated relative to untreated controls (n = 3).

Biofilm inhibition assay. *S. aureus* (ATCC 25923, 10^6 CFU/mL) and *P. aeruginosa* (PAO1, 10^5 CFU/mL) were suspended in MHB2 medium, and 1.5 mL of each bacterial solution was dispensed into 24-well plates. TM1S-incorporated hydrogels (200 μL) were added to each well and incubated at 37 °C for 24 h to allow biofilm formation. After incubation, hydrogels and media were removed, and the wells were gently rinsed twice with PBS to eliminate planktonic bacteria. Biofilms were fixed with 2 mL of methanol for 15 min, stained with 1.5 mL of 1% (w/v) crystal violet for 5 min, and washed thoroughly with PBS. The bound dye was solubilized in 33% (w/v) acetic acid, and absorbance at 590 nm was measured on a microplate reader to quantify biofilm biomass. For Live/Dead imaging, biofilms were cultured on sterile circular coverslips (12 mm diameter) placed in 24-well plates with 200 μL of TM1S-incorporated hydrogel. After 24 h of incubation, hydrogels and media were removed, and coverslips were rinsed twice with PBS. Biofilms were stained with SYTO 9 and PI for 30 min in the dark, followed by two PBS washes. Samples were then fixed with 4% paraformaldehyde, rinsed with

PBS, and observed by CLSM (λ_{ex} = 488 nm for SYTO 9; λ_{ex} = 561 nm for PI). For image-based analysis, z-stacked CLSM images were processed using ImageJ software to determine the pixel area corresponding to live (SYTO 9) and dead (PI) bacterial signals.

To visualize biofilm EPS and measure biofilm thickness, samples were stained with concanavalin A (ConA)-Alexa Fluor 488 conjugate followed by CLSM analysis. Biofilms were cultured on sterile circular coverslips (10 mm diameter) placed in 24-well plates containing 200 μL of TM1S-incorporated hydrogel. After 24 h of incubation, the hydrogels and media were removed, and coverslips were rinsed twice with PBS. Biofilms were then stained with the ConA-Alexa Fluor 488 conjugate and PI for 30 min in the dark, followed by two PBS washes. Samples were fixed with 4% paraformaldehyde, rinsed with PBS, and observed by CLSM (λ_{ex} = 488 nm for ConA-Alexa Fluor 488 conjugate; λ_{ex} = 561 nm for PI). Biofilm thickness was quantified by generating surfaces from the Alexa Fluor™ 488 signal within the z-stack datasets. 3D particle coordinates exported from Imaris 9.7 (Bitplane AG, Switzerland) were used for analysis. The XY plane was partitioned into square bins of 5 μm × 5 μm. For each XY bin, particles assigned to that bin were identified, and local thickness (T) was defined as the axial extent of the particles within the bin, calculated as:

$$T\ (xi, yj) = \max(Z) - \min(Z)$$

To suppress unstable estimates from sparsely sampled regions, bins containing fewer than 5 particles were excluded. The resulting set of local thickness values was summarized by mean, standard deviation, median, minimum, maximum, and the 10th and 90th percentiles. Thickness distributions were visualized as histograms of local thickness across all retained bins. All analyses were performed using a fixed bin size (5 μm) and minimum-particle threshold ($n \geq 5$) across all conditions and channels to ensure direct comparability.

For SEM analysis, biofilms treated with TM1S-incorporated hydrogels were grown on coverslips and fixed with 4% paraformaldehyde. The fixed samples were then washed twice with PBS, dehydrated through an ethanol gradient (30, 50, 70, and 100%), and then subjected to critical point drying. The morphology of the biofilm matrix and bacterial cells was visualized by SEM.

Swelling Ratio Measurement. Swelling ratio measurements were performed to evaluate the swelling behavior of the hydrogels under physiological conditions. Lyophilized hydrogel samples were first weighed to obtain the dry mass (W_d), which served as the reference state (0 min, 0%). The samples were then immersed in 1× PBS and incubated at room temperature. At predetermined time points, the hydrogels were removed, gently blotted with filter paper to eliminate excess surface liquid, and weighed to determine the swollen mass (W_s). The swelling ratio was calculated using the following equation: $(W_s - W_d)/W_d \times 100$. All measurements were performed in triplicate ($n = 3$) to ensure reproducibility.

Compressive Stress–Strain Measurements. Compressive stress–strain properties were characterized using a universal testing machine (Instron 5966, USA) equipped with a 500 N load cell under ambient conditions. Cylindrical hydrogel specimens (10 mm in diameter) were prepared for all compression measurements. Prior to testing, the initial height of each specimen was measured to define the reference dimension for strain calculations. In both monotonic compression and loading–unloading cyclic tests, a constant nominal strain rate of 10% min^{-1} was applied with respect to the original specimen height.

Statistical analysis and reproducibility. All data are presented as mean ± SD from at least three independent experiments, unless otherwise specified. Statistical analyses were performed using Origin 2022 (OriginLab Corporation, USA). Welch's t-test was used as indicated in the figure captions. Statistical significance was defined at $p < 0.05$ (*), $p < 0.01$ (**), and $p < 0.001$ (***).

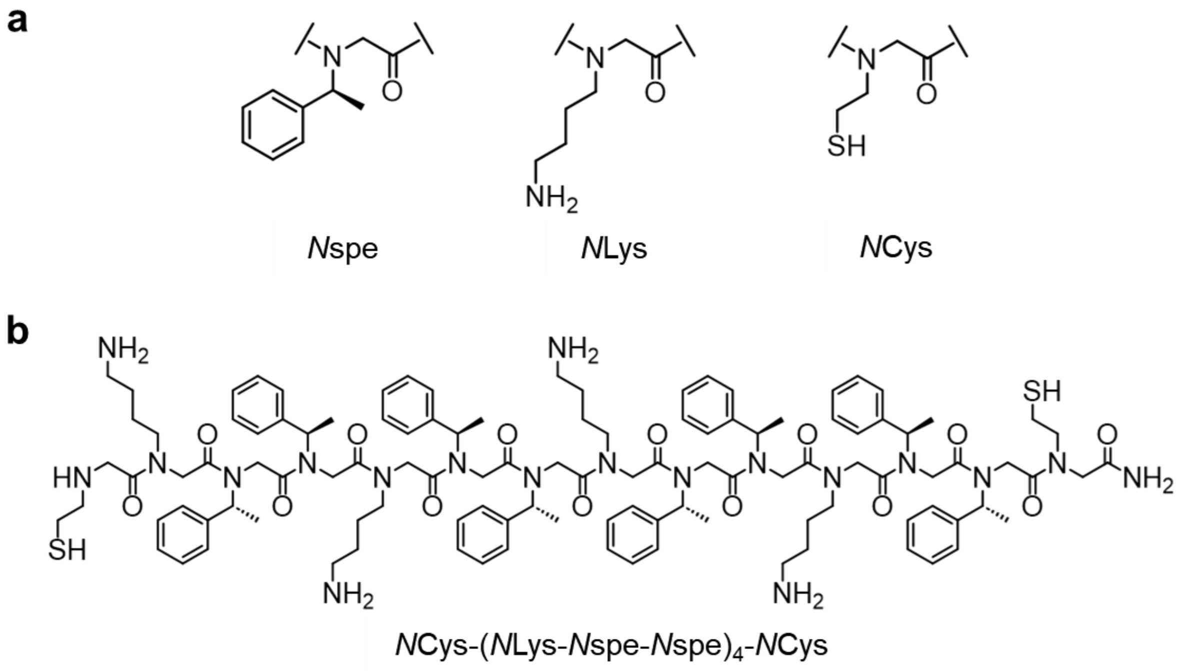

Figure S1. Chemical structures of (a) peptoid monomers and (b) TM1S.

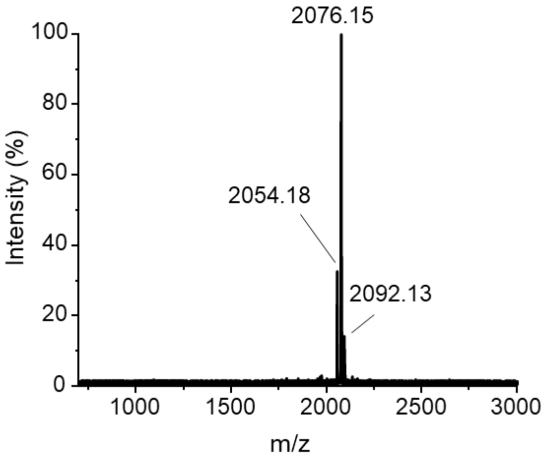

Compound	Calculated mass			Observed mass		
	$[M+H]^+$	$[M+Na]^+$	$[M+K]^+$	$[M+H]^+$	$[M+Na]^+$	$[M+K]^+$
**TM1S**	2053.13	2075.13	2091.13	2054.18	2076.15	2092.13

Figure S2. MALDI-TOF mass spectrum of TM1S.

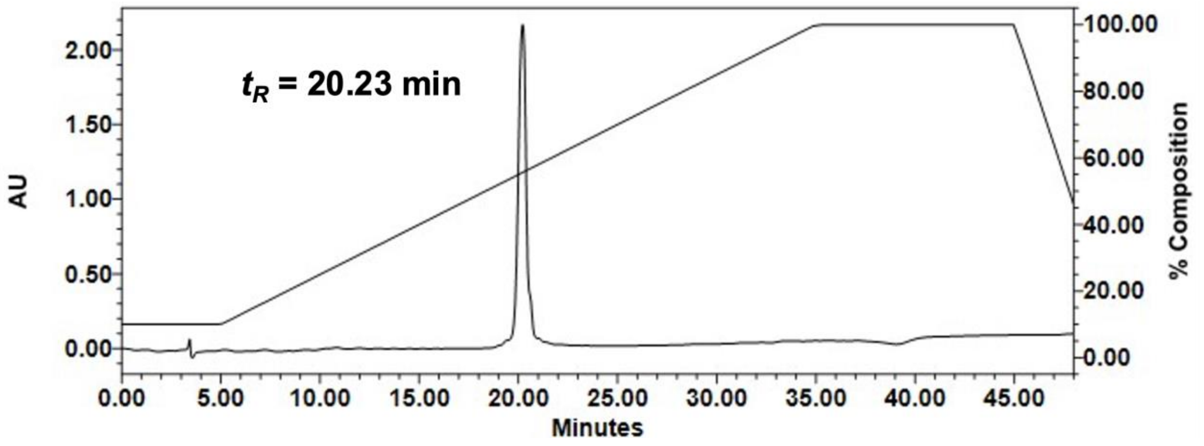

Figure S3. HPLC chromatogram of TM1S with UV detection at 220 nm.

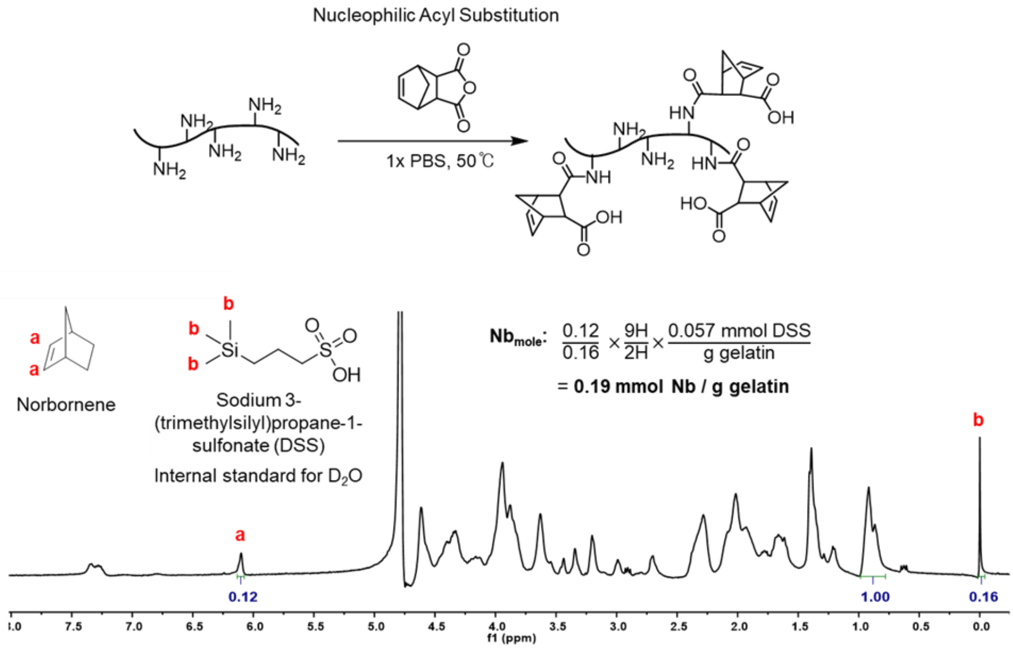

Figure S4. Synthesis scheme of norbornene (Nb)-functionalized gelatin (Gel-Nb) and determination of degree of Nb functionalization by ^{1}H NMR spectrum.

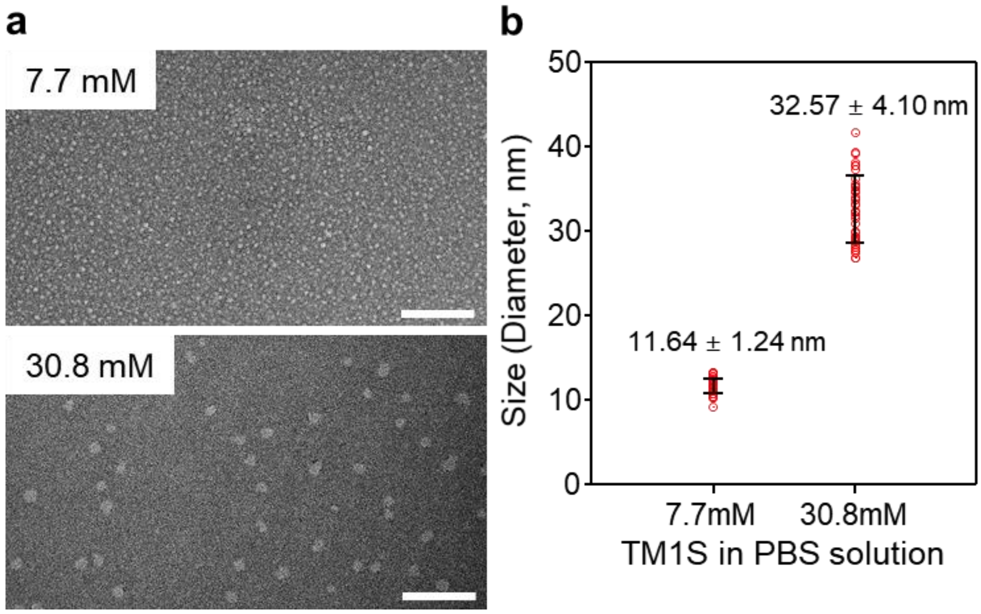

Figure S5. (a) Representative TEM images of TM1S assemblies at 7.7 mM and 30.8 mM in PBS (1×, pH 7.4). The scale bars represent 200 nm. (b) Statistical analysis of particle diameters at each concentration. Individual particle measurements are plotted as open circles (mean ± SD, n = 40).

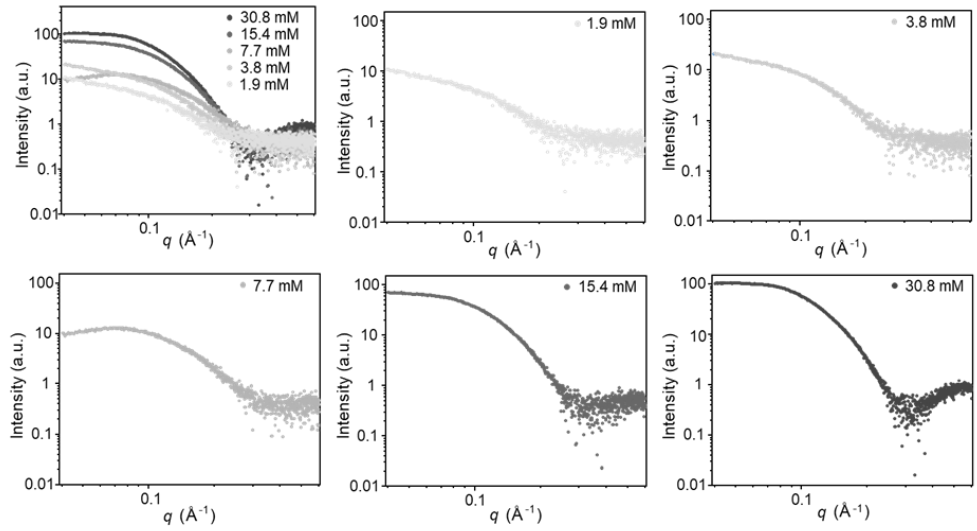

Figure S6. SAXS spectra of TM1S assemblies in PBS at various TM1S concentrations.

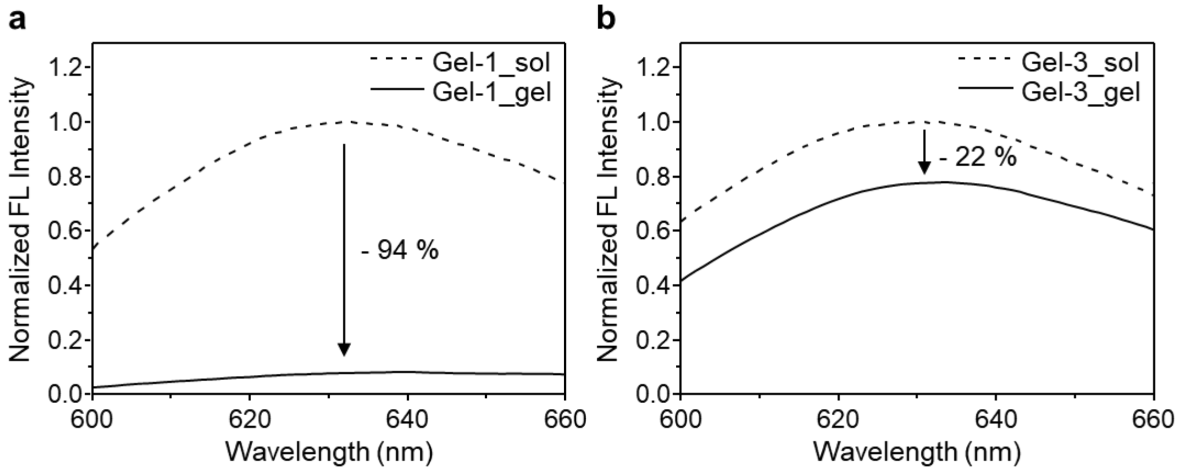

Figure S7. Normalized fluorescence spectra of Nile Red in TM1S-containing systems before (sol) and after (gel) gelation for (a) Gel-1 and (b) Gel-3. Fluorescence intensities were normalized relative to the respective pre-gelation state to highlight the magnitude of intensity changes by gelation.

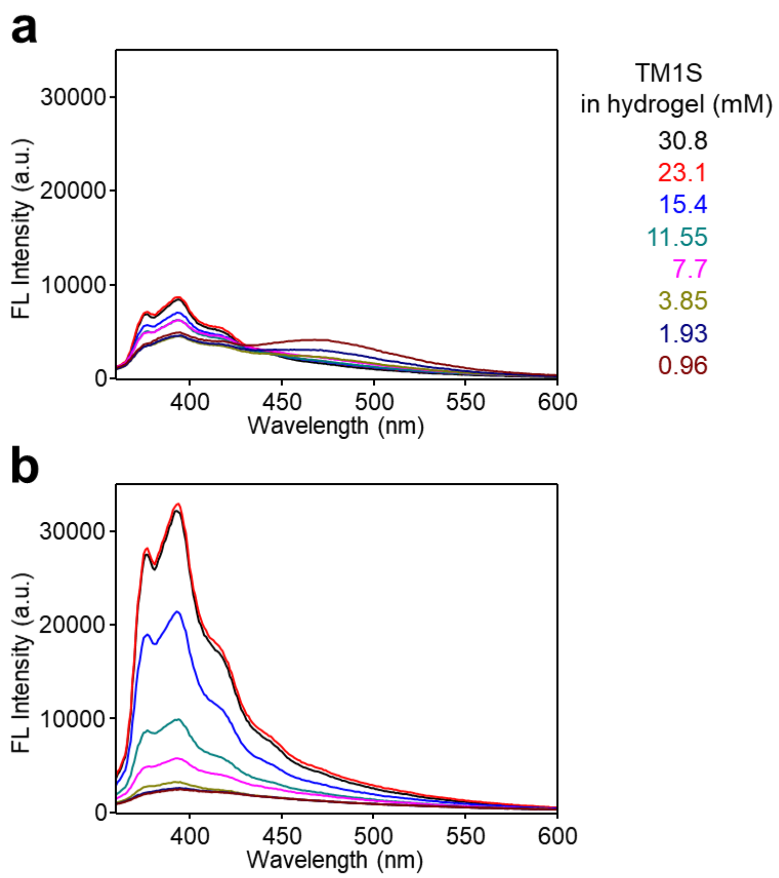

Figure S8. Pyrene fluorescence spectra of TM1S-incorporated hydrogels at varying TM1S concentrations. (a) Fluorescence emission spectra before gelation and (b) after gelation, recorded at an excitation wavelength of 335 nm. Depending on TM1S concentration, gelation leads to an increase or decrease in pyrene fluorescence intensity, indicating concentration-dependent changes in the local environment of pyrene.

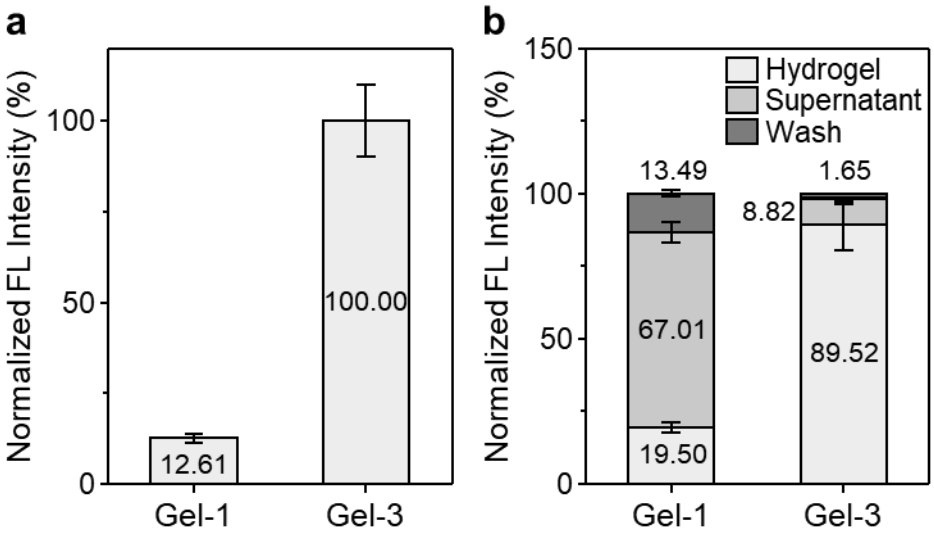

Figure S9. (a) Relative and (b) normalized fluorescence intensities of fluorescein conjugated bovine serum albumin (FITC-BSA) absorbed on TM1S-incorporated hydrogel surfaces at varying TM1S concentrations (mean ± SD, n = 3).

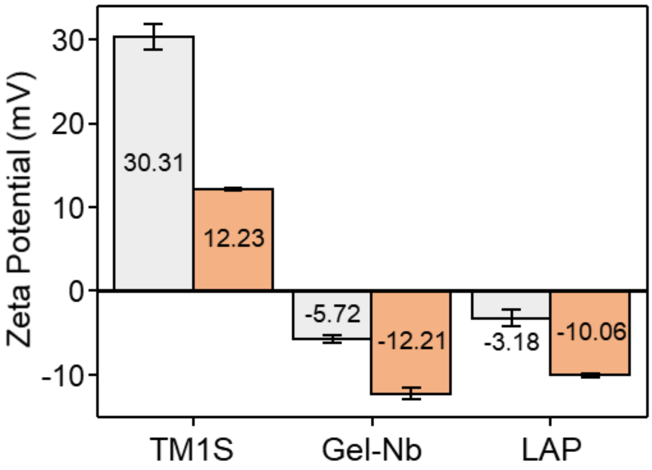

Figure S10. Zeta potentials of TM1S-incorporated hydrogel components (0.1 wt%), measured in 1× PBS buffer (gray) and 10 mM KCl solution (red) (mean ± SD, n =3).

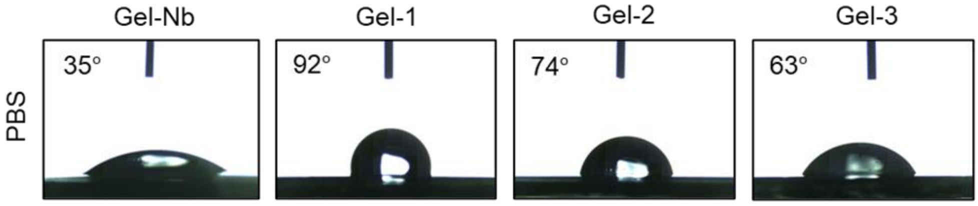

Figure S11. Contact angle images of TM1S-incorporated hydrogels using 3 μL PBS as a droplet.

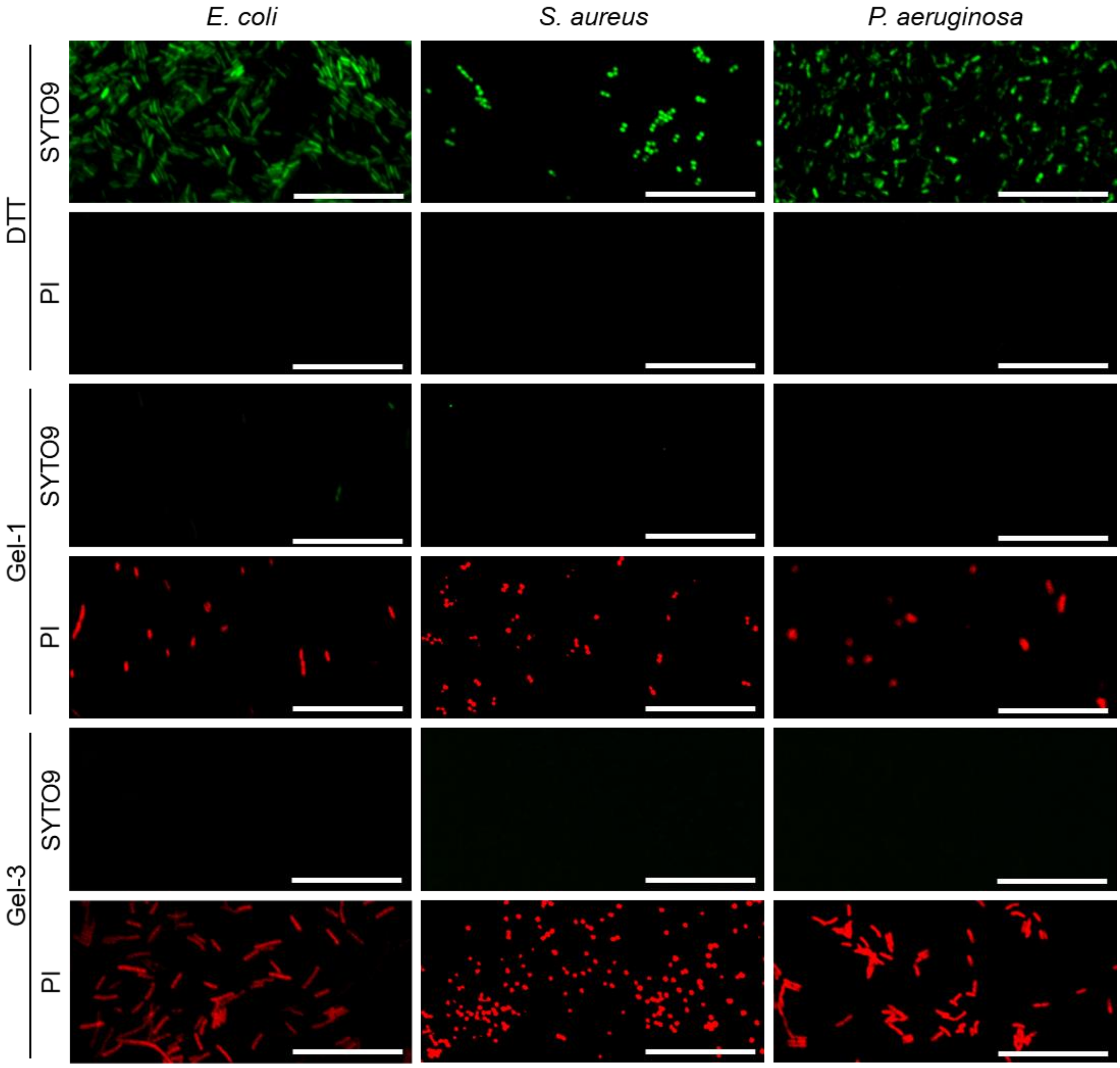

Figure S12. Individual fluorescence channels corresponding to Figure 3e. Live bacteria were stained with SYTO9 (green) and dead bacteria with propidium iodide (PI, red). The scale bar represents 20 μm.

Materials	MIC (μM)			MBC (μM)		
	E. coli	*S. aureus*	*P. aeruginosa*	*E. coli*	*S. aureus*	*P. aeruginosa*
	**ATCC 25922**	**ATCC 25923**	**PAO1**	**ATCC 25922**	**ATCC 25923**	**PAO1**
DTT	>1000	>1000	>1000	>1000	>1000	>1000
TM1S	62.5	7.8125	31.25	62.5	15.625	62.5
LAP	>1000	>1000	>1000	>1000	>1000	>1000
GelNb	>1000	>1000	>1000	>1000	>1000	>1000
TM1S H.	125	7.8125	31.25	125	31.25	125
DTT H.	>1000	>1000	>1000	>1000	>1000	>1000

Table S1. MIC and MBC values of hydrogel components against *E. coli*, *S. aureus* and *P. aeruginosa.* The tested concentration range was 0.5–1000 μM using a two-fold serial dilution (n = 3). H. denotes the hydrogel precursor solution prior to UV irradiation, used as a control to evaluate the influence of GelNb and LAP.

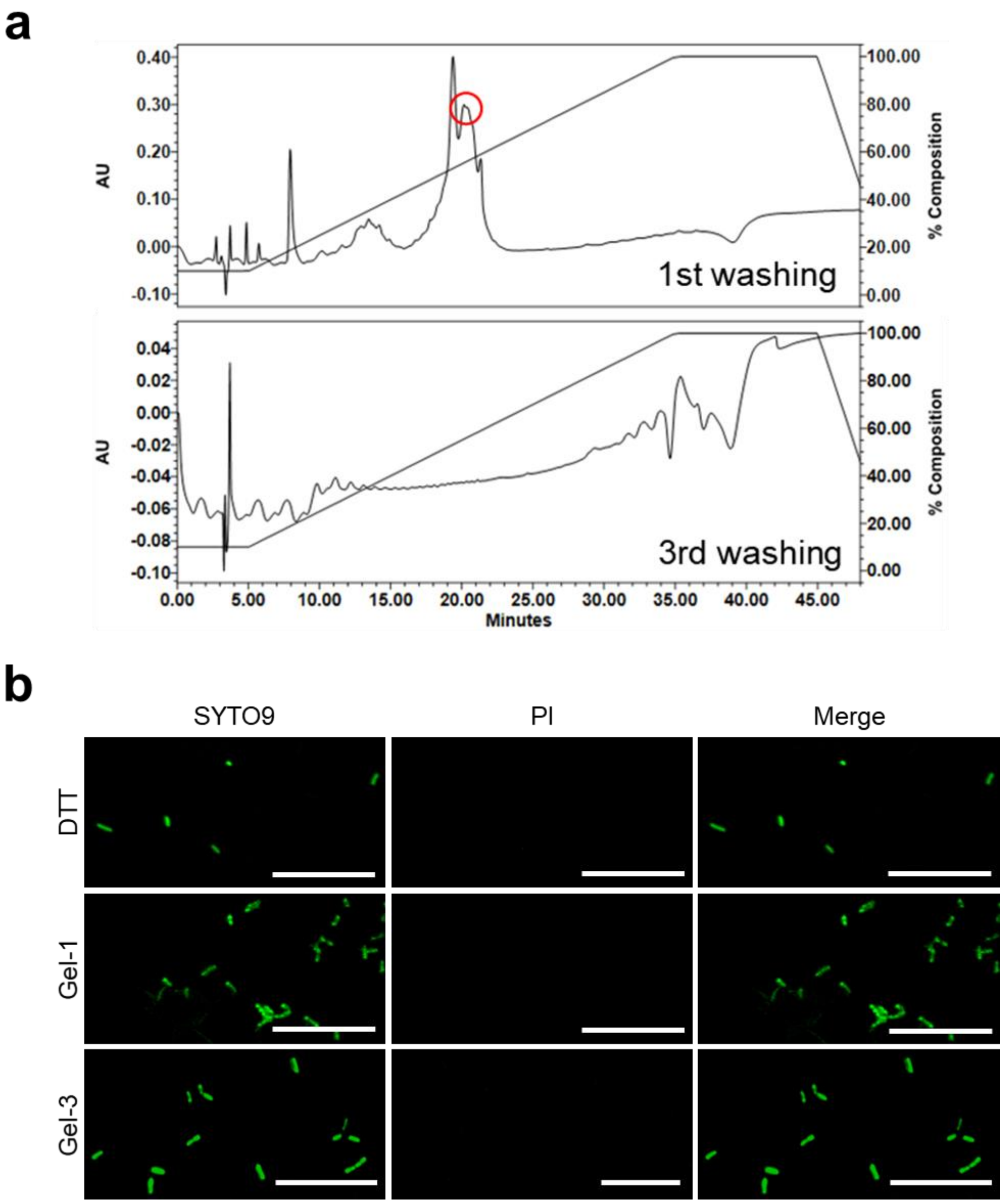

Figure S13. Validation of TM1S immobilization in TM1S-incorporated hydrogels. (a) HPLC chromatograms of supernatants collected after the first and third washing steps of Gel-3. A distinct TM1S peak was observed after the first wash; however, no characteristic peak was detectable after the third wash, indicating that the concentration of leaked TM1S decreased below the detection limit through repeated washing. (b) Representative fluorescence images of Live/Dead-stained *E. coli* treated with eluates derived from TM1S-incorporated hydrogels after 6 h of incubation. The scale bar represents 20 µm.

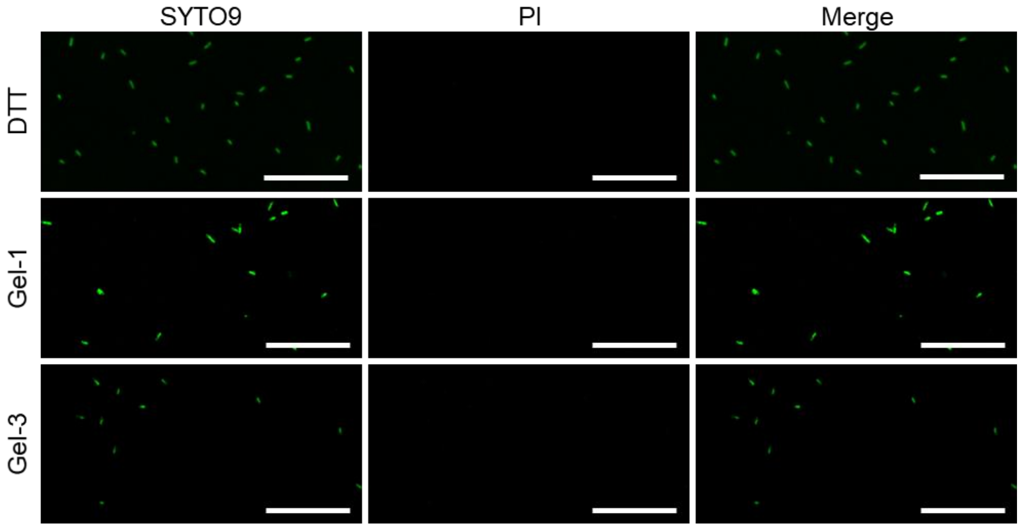

Figure S14. Fluorescence images of Live/Dead-stained *E. coli* in the supernatant collected after 6 h incubation on TM1S-incorporated hydrogels. The scale bar represents 20 μm.

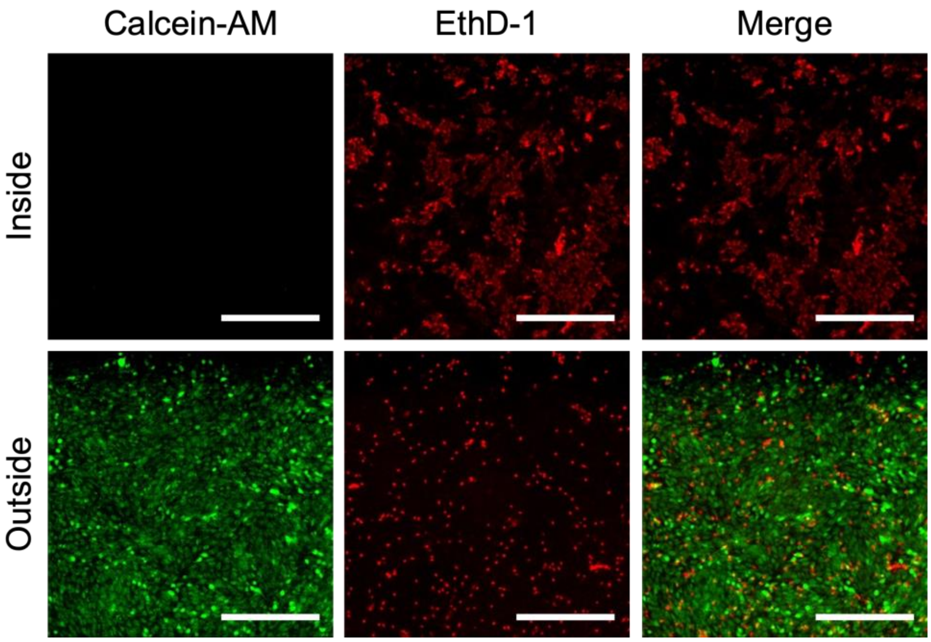

Figure S15. Cytotoxicity of TM1S-incorporated hydrogels at the Gel-3 formulation. Fluorescence images of C2C12 myoblasts treated with Gel-3, showing regions with (inside) and without (outside) direct contact. The scale bar represents 20 μm.

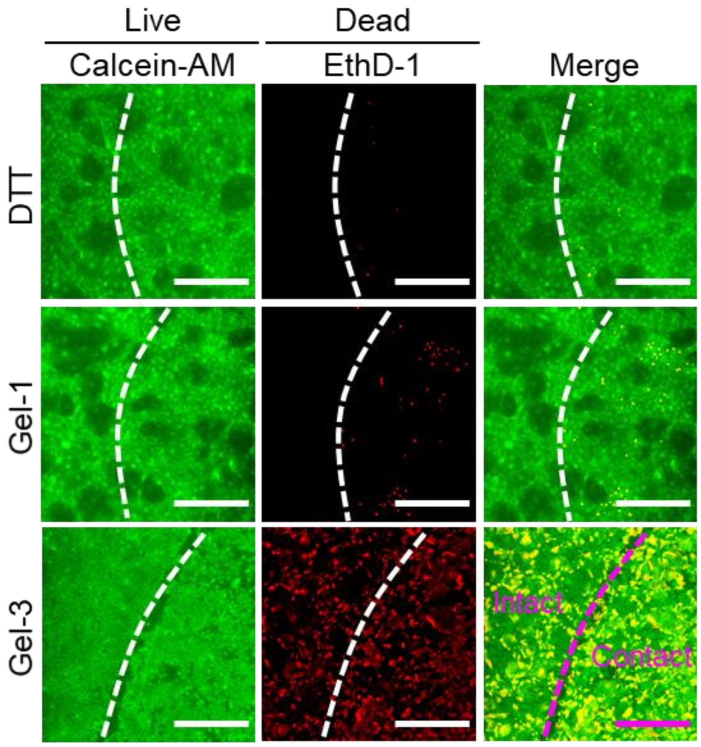

Figure S16. Live/Dead-stained images of HaCaT cells after incubation with TM1S-incorporated hydrogels to evaluate cytotoxicity. Scale bars represent 200 μm.

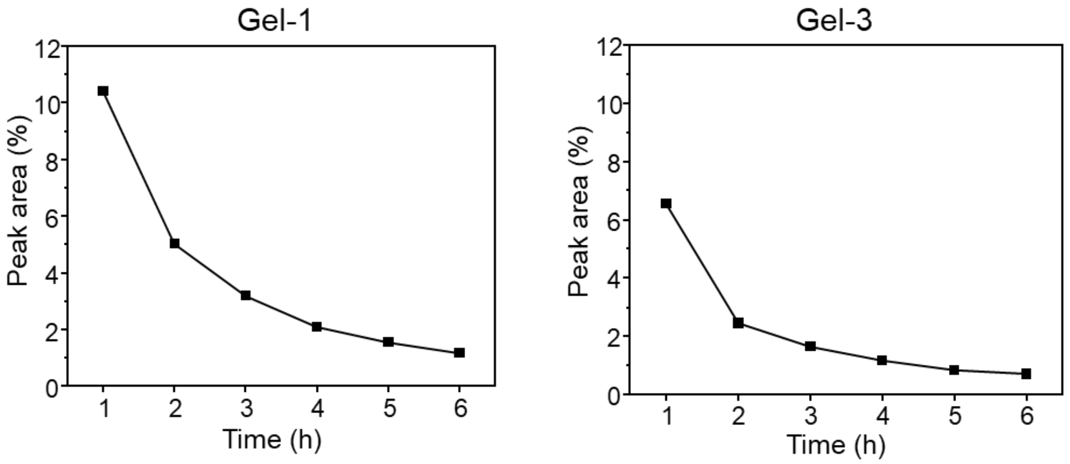

Figure S17. Relative peak area of released TM1S from TM1S-incorporated hydrogels using PBS as washing solution (200 µL of hydrogel in 5 mL of PBS). At each time point, 1 mL of supernatant was collected for analytical HPLC analysis and replaced with 1 mL of fresh PBS for the subsequent washing step. The experiment was conducted at 37 °C in a shaking incubator. Data are shown for a single experiment (n = 1).

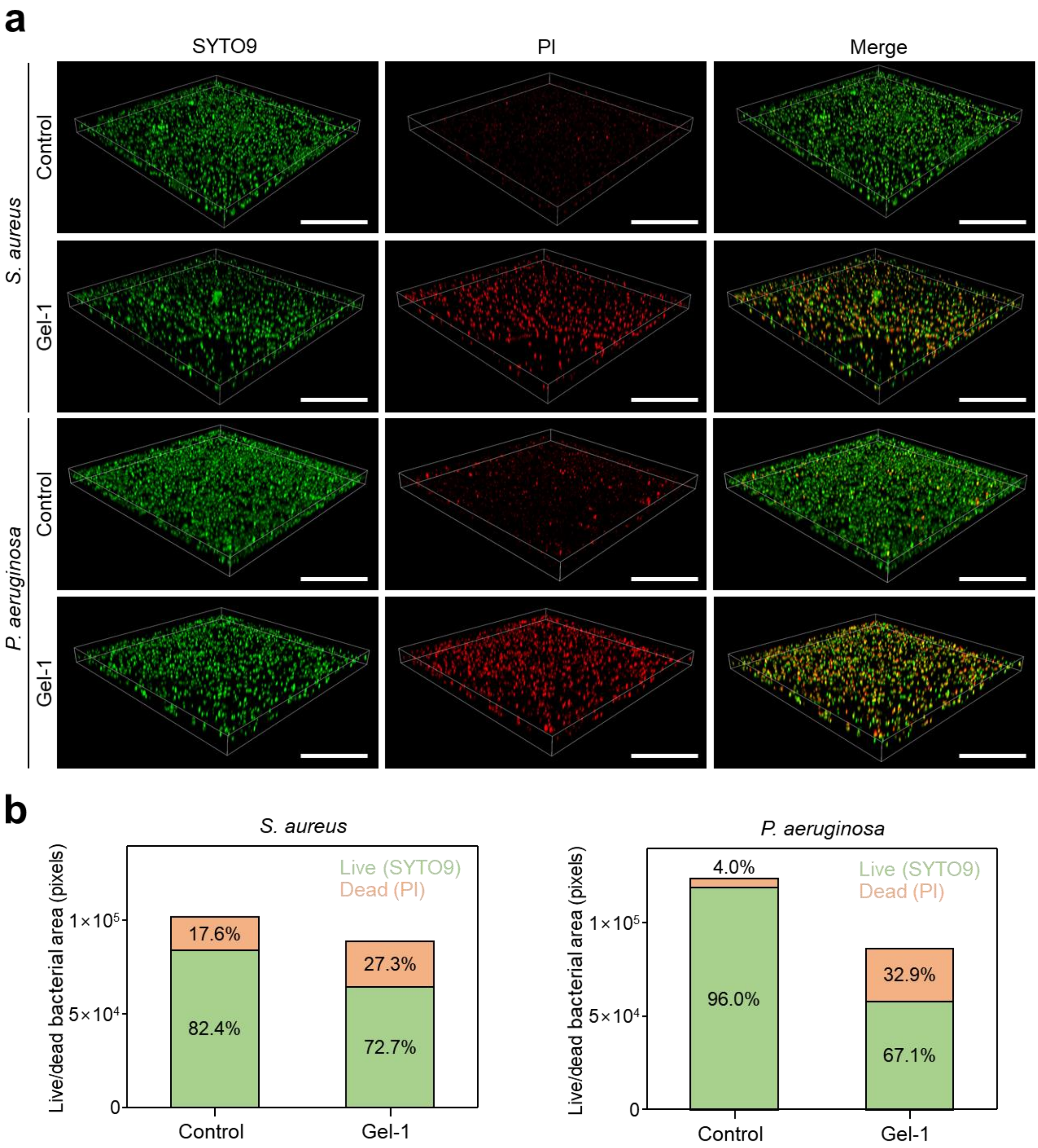

Figure S18. Quantitative analysis of bacterial viability in *S. aureus* and *P. aeruginosa* biofilms treated with Gel-1. (a) Representative Z-projected CLSM images of biofilms after treatment, stained with SYTO9 (green, live) and propidium iodide (PI, red, dead). Scale bars represent 20 μm. (b) Quantification of live and dead bacterial areas derived from pixel counts of Z-projected images. The stacked bars represent the total pixel areas, with the internal percentages indicating the relative proportions of live (SYTO9-positive) and dead (PI-positive) bacteria.

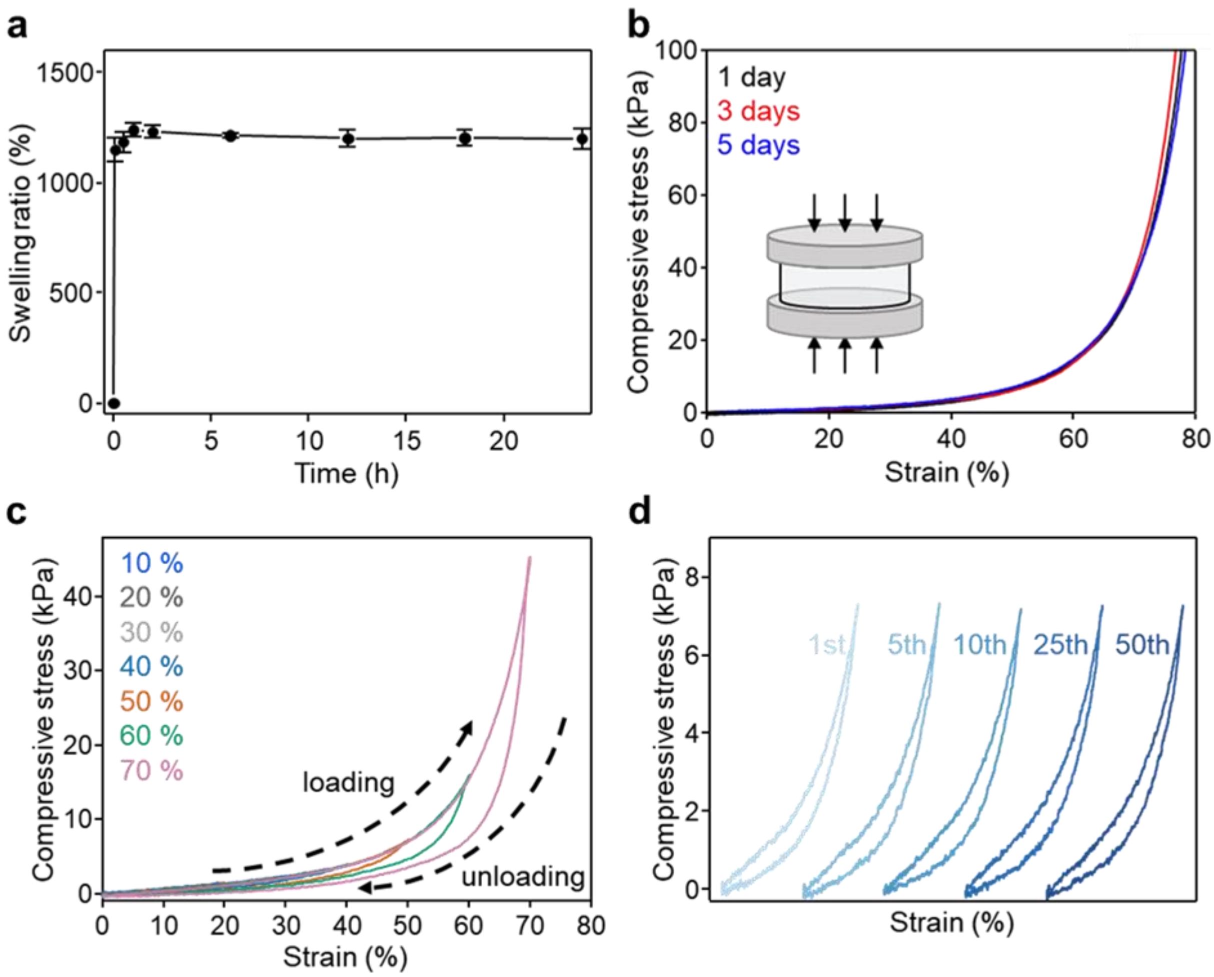

Figure S19. Mechanical stability and deformation behavior of Gel-1 under physiologically relevant conditions. (a) Time-dependent swelling ratio of Gel-1 in 1× PBS (mean ± SD, n = 3). The swelling ratio was calculated relative to the initial lyophilized state (0 min = 0%), showing rapid swelling followed by a stable equilibrium plateau within 2 h. (b) Compressive stress–strain curves of Gel-1 recorded after incubation in 1× PBS for 1, 3, and 5 days, demonstrating the preservation of mechanical integrity and resistance to hydration degradation under prolonged swelling. (c) Compressive stress–strain responses of Gel-1 under increasing maximum compressive strains (10–70%), indicating large-scale, fully recoverable deformation without structural failure. (d) Representative loading–unloading cyclic compression profiles of Gel-1 over 50 consecutive cycles at 10% strain, showing minimal hysteresis and high fatigue resistance.

References

(1) Zuckermann, R. N.; Kerr, J. M.; Kent, S. B.; Moos, W. H. Efficient method for the preparation of peptoids [oligo (N-substituted glycines)] by submonomer solid-phase synthesis. *J. Am. Chem. Soc.* **1992**, *114* (26), 10646-10647.

(2) Kirshenbaum, K.; Barron, A. E.; Goldsmith, R. A.; Armand, P.; Bradley, E. K.; Truong, K. T. V.; Dill, K. A.; Cohen, F. E.; Zuckermann, R. N. Sequence-specific polypeptoids: A diverse family of heteropolymers with stable secondary structure. *Proc. Natl. Acad. Sci. U. S. A.* **1998**, *95* (8), 4303-4308.

(3) Halkes, K. M.; Carvalho de Souza, A.; Maljaars, C. E. P.; Gerwig, G. J.; Kamerling, J. P. A facile method for the preparation of gold glyconanoparticles from free oligosaccharides and their applicability in carbohydrate-protein interaction studies. *Eur. J. Org. Chem.* **2005**, (17), 3650-3659.

(4) Schröder, T.; Schmitz, K.; Niemeier, N.; Balaban, T. S.; Krug, H. F.; Schepers, U.; Bräse, S. Solid-phase synthesis, bioconjugation, and toxicology of novel cationic oligopeptoids for cellular drug delivery. *Bioconjug. Chem.* **2007**, *18* (2), 342-354.

(5) Chen, Y.-C.; Lin, R.-Z.; Qi, H.; Yang, Y.; Bae, H.; Melero-Martin, J. M.; Khademhosseini, A. Functional human vascular network generated in photocrosslinkable gelatin methacrylate hydrogels. *Adv. Funct. Mater.* **2012**, *22* (10), 2027-2039.

(6) Mũnoz, Z.; Shih, H.; Lin, C.-C. Gelatin hydrogels formed by orthogonal thiol–norbornene photochemistry for cell encapsulation. *Biomater. Sci.* **2014**, *2* (8), 1063-1072.

(7) Claaßen, C.; Claaßen, M. H.; Truffault, V.; Sewald, L.; Tovar, G. E. M.; Borchers, K.; Southan, A. Quantification of substitution of gelatin methacryloyl: Best practice and current pitfalls. *Biomacromolecules* **2018**, *19* (1), 42-52.

(8) Kim, K.-W.; Kim, J.; Yun, Y. D.; Ahn, H.; Min, B.; Kim, N. H.; Rah, S.; Kim, H.-Y.; Lee, C.-S.; Seo, I. D.; Lee, W.-W.; Choi, H. J; Jin, K. S. Small-angle X-ray scattering beamline BL4C SAXS at Pohang light source II. *Biodesign* **2017**, *5* (1), 24-29.

(9) Lee, M. J.; Shrotriya, D. R.; Espinosa-Marzal, R. M. Responsiveness of charged double network hydrogels to ionic environment. *Adv. Funct. Mater.* **2024**, *34* (37), 2402279.
